# The International Limits and Population at Risk of *Plasmodium vivax* Transmission in 2009

**DOI:** 10.1371/journal.pntd.0000774

**Published:** 2010-08-03

**Authors:** Carlos A. Guerra, Rosalind E. Howes, Anand P. Patil, Peter W. Gething, Thomas P. Van Boeckel, William H. Temperley, Caroline W. Kabaria, Andrew J. Tatem, Bui H. Manh, Iqbal R. F. Elyazar, J. Kevin Baird, Robert W. Snow, Simon I. Hay

**Affiliations:** 1 Spatial Ecology and Epidemiology Group, Department of Zoology, University of Oxford, Oxford, United Kingdom; 2 Biological Control and Spatial Ecology, Université Libre de Bruxelles, CP160/12, Brussels, Belgium; 3 Malaria Public Health and Epidemiology Group, Centre for Geographic Medicine, KEMRI - University of Oxford - Wellcome Trust Collaborative Programme, Nairobi, Kenya; 4 Department of Geography, University of Florida, Gainesville, Florida, United States of America; 5 Emerging Pathogens Institute, University of Florida, Gainesville, Florida, United States of America; 6 Oxford University Clinical Research Unit, Bach Mai Hospital, National Institute of Infectious and Tropical Diseases, Ha Noi, Vietnam; 7 Eijkman-Oxford Clinical Research Unit, Jakarta, Indonesia; 8 Centre for Tropical Medicine, Nuffield Department of Clinical Medicine, Oxford University, Oxford, United Kingdom; 9 Centre for Tropical Medicine, Nuffield Department of Clinical Medicine, University of Oxford, CCVTM, Oxford, United Kingdom; New York University School of Medicine, United States of America

## Abstract

**Background:**

A research priority for *Plasmodium vivax* malaria is to improve our understanding of the spatial distribution of risk and its relationship with the burden of *P. vivax* disease in human populations. The aim of the research outlined in this article is to provide a contemporary evidence-based map of the global spatial extent of *P. vivax* malaria, together with estimates of the human population at risk (PAR) of any level of transmission in 2009.

**Methodology:**

The most recent *P. vivax* case-reporting data that could be obtained for all malaria endemic countries were used to classify risk into three classes: malaria free, unstable (<0.1 case per 1,000 people per annum (p.a.)) and stable (≥0.1 case per 1,000 p.a.) *P. vivax* malaria transmission. Risk areas were further constrained using temperature and aridity data based upon their relationship with parasite and vector bionomics. Medical intelligence was used to refine the spatial extent of risk in specific areas where transmission was reported to be absent (e.g., large urban areas and malaria-free islands). The PAR under each level of transmission was then derived by combining the categorical risk map with a high resolution population surface adjusted to 2009. The exclusion of large Duffy negative populations in Africa from the PAR totals was achieved using independent modelling of the gene frequency of this genetic trait. It was estimated that 2.85 billion people were exposed to some risk of *P. vivax* transmission in 2009, with 57.1% of them living in areas of unstable transmission. The vast majority (2.59 billion, 91.0%) were located in Central and South East (CSE) Asia, whilst the remainder were located in America (0.16 billion, 5.5%) and in the Africa+ region (0.10 billion, 3.5%). Despite evidence of ubiquitous risk of *P. vivax* infection in Africa, the very high prevalence of Duffy negativity throughout Central and West Africa reduced the PAR estimates substantially.

**Conclusions:**

After more than a century of development and control, *P. vivax* remains more widely distributed than *P. falciparum* and is a potential cause of morbidity and mortality amongst the 2.85 billion people living at risk of infection, the majority of whom are in the tropical belt of CSE Asia. The probability of infection is reduced massively across Africa by the frequency of the Duffy negative trait, but transmission does occur on the continent and is a concern for Duffy positive locals and travellers. The final map provides the spatial limits on which the endemicity of *P. vivax* transmission can be mapped to support future cartographic-based burden estimations.

## Author Summary

Growing evidence shows that *Plasmodium vivax* malaria is clinically less benign than has been commonly believed. In addition, it is the most widely distributed species of human malaria and is likely to cause more illness in certain regions than the more extensively studied *P. falciparum* malaria. Understanding where *P. vivax* transmission exists and measuring the number of people who live at risk of infection is a fundamental first step to estimating the global disease toll. The aim of this paper is to generate a reliable map of the worldwide distribution of this parasite and to provide an estimate of how many people are exposed to probable infection. A geographical information system was used to map data on the presence of *P. vivax* infection and spatial information on climatic conditions that impede transmission (low ambient temperature and extremely arid environments) in order to delineate areas where transmission was unlikely to take place. This map was combined with population distribution data to estimate how many people live in these areas and are, therefore, exposed to risk of infection by *P. vivax* malaria. The results show that 2.85 billion people were exposed to some level of risk of transmission in 2009.

## Introduction

The bulk of the global burden of human malaria is caused by two parasites: *Plasmodium falciparum* and *P. vivax*. Existing research efforts have focussed largely on *P. falciparum* because of the mortality it causes in Africa [1], [2]. This focus is increasingly regarded as untenable [3]–[6] because the following factors indicate that the public health importance of *P. vivax* may be more significant than traditionally thought: i) *P. vivax* has a wider geographical range, potentially exposing more people to risk of infection [7], [8]; ii) it is less amenable to control [9], [10]; and, most importantly, iii) infections with *P. vivax* can cause severe clinical syndromes [5], [11]–[16].

A key research priority for *P. vivax* malaria is to improve the basic understanding of the geographical distribution of risk, which is needed for adequate burden estimation [6]. Recent work by the Malaria Atlas Project (MAP; www.map.ox.ac.uk) [17] has shown *P. falciparum* malaria mapping to be a fundamental step in understanding the epidemiology of the disease at the global scale [18], [19], in appraising the equity of global financing for control [20] and in forming the basis for burden estimation [21], [22]. The benefits of a detailed knowledge of the spatial distribution of *P. vivax* transmission, and its clinical burden within these limits, are identical to those articulated for *P. falciparum*: establishing a benchmark against which control targets may be set, budgeted and monitored. Such maps do not exist for *P. vivax*, making any strategic planning problematic. In addition, information about the global extent of *P. vivax* transmission and population at risk (PAR) is crucial for many nations that are re-evaluating their prospects for malaria elimination [23], [24].

This paper documents the global spatial limits of *P. vivax* malaria using a combination of national case-reporting data from health management information systems (HMIS), biological rules of transmission exclusion and medical intelligence combined in a geographical information system. The output is an evidence-based map from which estimates of PAR are derived. The resulting map also provides the global template in which contemporary *P. vivax* endemicity can be estimated and it contributes to a cartographic basis for *P. vivax* disease burden estimation.

## Methods

### Analyses Outline

**Figure 1:**
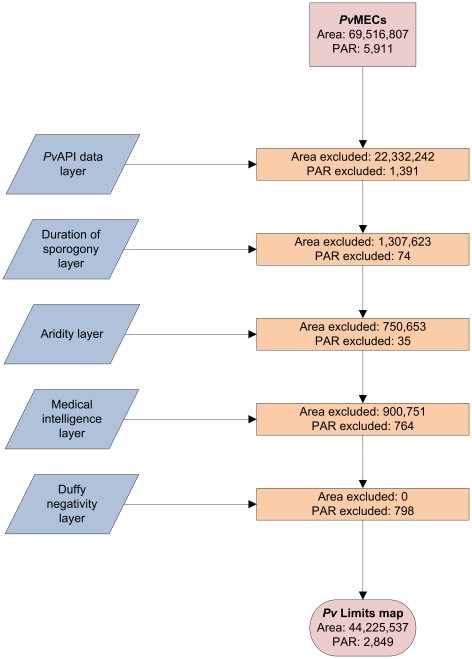
Flow chart of the various data and exclusion layers used to derive the final map. The pink rectangle denotes the surface area and populations of *Pv*MECs, whilst the pink ovoid represents the resulting trimmed surface area and PAR after the exclusion of risk by the various input layers, denoted by the blue rhomboids. Orange rectangles show area and PAR exclusions at each step to illustrate how these were reduced progressively. The sequence in which the exclusion layers are applied does not affect the final PAR estimates.

A schematic overview of the analyses is presented in Figure 1. Briefly, *P. vivax* malaria endemic countries (*Pv*MECs) were first identified and the following layers were progressively applied within a geographical information system to constrain risk areas and derive the final *P. vivax* spatial limits map: i) a *P. vivax* annual parasite incidence (*Pv*API) data layer; biological exclusion layers comprising of ii) temperature and iii) aridity data layers; iv) a medical intelligence exclusion layer; and v) a predicted Duffy negativity layer. A detailed description of these steps follows.

### Identifying *Pv*MECs

Those countries that currently support *P. vivax* transmission were first identified. The primary sources for defining national risk were international travel and health guidelines [25], [26] augmented with national survey information, pertinent published sources and personal communication with malariologists. Nations were grouped into three regions, as described elsewhere [19]: i) America; ii) Africa, Saudi Arabia and Yemen (Africa+); and iii) Central and South East (CSE) Asia. To further resolve PAR estimates, the CSE Asia region was sub-divided into West Asia, Central Asia and East Asia (Protocol S1).

### Mapping case-reporting data

Methods described previously for mapping the global spatial limits of *P. falciparum* malaria [18] were used to constrain the area defined at risk within the *Pv*MECs using *Pv*API data (the number of confirmed *P. vivax* malaria cases reported per administrative unit per 1,000 people per annum (p.a.)). The *Pv*API data were obtained mostly through personal communication with individuals and institutions linked to malaria control in each country (Protocol S1). The format in which these data were available varied considerably between countries. Ideally, the data would be available by administrative unit and by year, with each record presenting the estimated population for the administrative unit and the number of confirmed autochthonous malaria cases by the two main parasite species (*P. falciparum* and *P. vivax*). This would allow an estimation of species-specific API. These requirements, however, were often not met. Population data by administrative unit were sometimes unavailable, in which cases these data were sourced separately or extrapolated from previous years. An additional problem was the lack of parasite species-specific case or API values. In such cases, a parasite species ratio was inferred from alternative sources and applied to provide an estimate of species-specific API. There was, thus, significant geographical variation in the ability to look at the relative frequency of these parasites between areas and this was not investigated further. Finally, although a differentiation between confirmed and suspected cases and between autochthonous and imported cases was often provided, whenever this was not available it was assumed that the cases in question referred to confirmed and autochthonous occurrences.

The aim was to collate data for the last four years of reporting (ideally up to 2009) at the highest spatial resolution available (ideally at the second administrative level (ADMIN2) or higher). A geo-database was constructed to archive this information and link it to digital administrative boundaries of the world available from the 2009 version of the Global Administrative Unit Layers (GAUL) data set, implemented by the Food and Agriculture Organization of the United Nations (FAO) within the EC FAO Food Security for Action Programme [27]. The *Pv*API data were averaged over the period available and were used to classify areas as malaria free, unstable (<0.1 case per 1,000 p.a.) or stable (≥0.1 case per 1,000 p.a.) transmission, based upon metrics advised during the Global Malaria Eradication Programme [28]–[30]. These data categories were then mapped using ArcMAP 9.2 (ESRI 2006).

### Biological masks of exclusion of risk

To further constrain risk within national territories, two “masks” of biological exclusion were implemented (Protocol S2). First, risk was constrained according to the relationship between temperature and the duration of sporogony, based upon parameters specific to *P. vivax* [31]. Synoptic mean, maximum and minimum monthly temperature records were obtained from 30-arcsec (∼1×1 km) spatial resolution climate surfaces [32]. For each pixel, these values were converted, using spline interpolation, to a continuous time series representing a mean temperature profile across an average year. Diurnal variation was represented by adding a sinusoidal component to the time series with a wavelength of 24 hours and the amplitude varying smoothly across the year determined by the difference between the monthly minimum and maximum values. For *P. vivax* transmission to be biologically feasible, a cohort of anopheline vectors infected with *P. vivax* must survive long enough for sporogony to complete within their lifetime. Since the rate of parasite development within anophelines is strongly dependent on ambient temperature, the time required for sporogony varies continuously as temperatures fluctuate across a year [31]. For each pixel, the annual temperature profile was used to determine whether any periods existed in the year when vector lifespan would exceed the time required for sporogony, and hence when transmission was not precluded by temperature. This was achieved via numerical integration whereby, for cohorts of vectors born at each successive 2-hour interval across the year, sporogony rates varying continuously as a function of temperature were used to identify the earliest time at which sporogony could occur. If this time exceeded the maximum feasible vector lifespan, then the cohort was deemed unable to support transmission. If sporogony could not complete for any cohort across the year, then the pixel was classified as being at zero risk. Vector lifespan was defined as 31 days since estimates of the longevity of the main dominant vectors [33] indicate that 99% of anophelines die in less than a month and, therefore, would be unable to support parasite development in the required time. The exceptions were areas that support the longer-lived *Anopheles sergentii* and *An. superpictus*, where 62 days were considered more appropriate (Protocol S2) [18].

The second mask was based on the effect of arid conditions on anopheline development and survival [34]. Limited surface water reduces the availability of sites suitable for oviposition and reduces the survival of vectors at all stages of their development through the process of desiccation [35]. The ability of adult vectors to survive long enough to contribute to parasite transmission and of pre-adult stages to ensure minimum population abundance is, therefore, dependent on the levels of aridity and species-specific resilience to arid conditions. Extremely arid areas were identified using the global GlobCover Land Cover product (ESA/ESA GlobCover Project, led by MEDIAS-France/POSTEL) [36]. GlobCover products are derived from data provided by the Medium Resolution Imaging Spectrometer (MERIS), on board the European Space Agency's (ESA) ENVIronmental SATellite (ENVISAT), for the period between December 2004 and June 2006, and are available at a spatial resolution of 300 meters [36]. The layer was first resampled to a 1×1 km grid using a majority filter, and all pixels classified as “bare areas” by GlobCover were overlaid onto the *Pv*API surface. The aridity mask was treated differently from the temperature mask to allow for the possibility of the adaptation of human and vector populations to arid environments [37]–[39]. A more conservative approach was taken, which down-regulated risk by one class. In other words, GlobCover's bare areas defined originally as at stable risk by *Pv*API were stepped down to unstable risk and those classified initially as unstable to malaria free.

### Medical intelligence modulation of risk

Medical intelligence contained in international travel and health guidelines [25], [26] was used to inform risk exclusion and down-regulation in specific urban areas and sub-national territories, which are cited as being free of malaria transmission (Protocol S3). Additional medical intelligence and personal communication with malaria experts helped identify further sub-national areas classified as malaria free in Cambodia, Vanuatu and Yemen. Specified urban areas were geo-positioned and their urban extents were identified using the Global Rural Urban Mapping Project (GRUMP) urban extents layer [40]. Rules of risk modulation within these urban extents were as follows: i) risk within urban extents falling outside the range of the urban vector *An. stephensi* [41] (Protocol S3) was excluded; ii) risk within urban areas inhabited by *An. stephensi* was down-regulated by one level from stable to unstable and from unstable to free (Protocol S3). Specified sub-national territories were classified as malaria free if not already identified as such by the *Pv*API layer and the biological masks. These territories were mapped using the GAUL data set [27].

### Duffy negativity phenotype

Since Duffy negativity provides protection against infection with *P. vivax* [42], a continuous map of the Duffy negativity phenotype was generated from a geostatistical model fully described elsewhere (Howes *et al.*, manuscript in preparation). The model was informed by a database of Duffy blood group surveys assembled from thorough searches of the published literature and supplemented with unpublished data by personal communication with relevant authors. Sources retrieved were added to existing Duffy blood group survey databases [43], [44]. The earliest inclusion date for surveys was 1950, when the Duffy blood group was first described [45].

To model the Duffy system and derive a global prediction for the frequency of the homozygous Duffy negative phenotype ([Fy(a-b-)], which is encoded by the homozygous FY*B*^ES^*/*B*^ES^* genotype), the spatially variable frequencies of the two polymorphic loci determining Duffy phenotypes were modelled: i) nucleotide −33 in the gene's promoter region, which defines positive/negative expression (T-33C); ii) the coding region locus (G125A) determining the antigen type expressed: Fy^a^ or Fy^b^ [46]. Due to the wide range of diagnostic methods used to describe Duffy blood types in recent decades, data were recorded in a variety of forms, each providing differing information about the frequency of variants at both loci. For example, some molecular studies sequenced only the gene's promoter region, and thus could not inform the frequency of the coding region variant; serological diagnoses only testing for the Fy^a^ antigen could not distinguish Fy^b^ from the Duffy negative phenotype. As part of the larger dataset, however, these incomplete data types can indirectly inform frequencies of negativity. Therefore, despite only requiring information about the promoter locus to model the negativity phenotype, variant frequencies at both polymorphic sites were modelled. This allowed the full range of information contained in the dataset to be used rather than just the subset specifically reporting Duffy negativity frequencies.

The model's general architecture and Bayesian framework will be described elsewhere (Howes *et al.*, manuscript in preparation). Briefly, the dataset of known values at fixed geographic locations was used to predict expression frequencies at each locus in all geographic sites where no data were available, thereby generating continuous global surfaces of the frequency of each variant. From the predicted frequency of the promoter region variant encoding null expression (-33C), a continuous frequency map of the Duffy negative population was derived.

### Estimating the population at risk of *P. vivax* transmission

The GRUMP *beta* version provides gridded population counts and population density estimates for the years 1990, 1995, and 2000, both adjusted and unadjusted to the United Nations' national population estimates [40]. The adjusted population counts for the year 2000 were projected to 2009 by applying national, medium variant, urban and rural-specific growth rates by country [47]. These projections were undertaken using methods described previously [48], but refined with urban growth rates being applied solely to populations residing within the GRUMP urban extents, while the rural growth rates were applied to the remaining population. This resulted in a 2009 population count surface of approximately 1×1 km spatial resolution, which was used to extract PAR figures. The PAR estimates in Africa were corrected for the presence of the Duffy negativity phenotype by multiplying the extracted population by [1 - frequency of Duffy negative individuals].

## Results

### *Plasmodium vivax* malaria endemic countries

A total of 109 potentially endemic countries and territories listed in international travel and health guidelines were identified [25], [26]. Ten of these countries: Algeria, Armenia, Egypt, Jamaica (*P. falciparum* only), Mauritius, Morocco, Oman, Russian Federation, Syrian Arab Republic and Turkmenistan have either interrupted transmission or are extremely effective at dealing with minor local outbreaks. These nations were not classified as *Pv*MECs and are all considered to be in the elimination phase by the Global Malaria Action Plan [24]. Additionally, four malaria endemic territories report *P. falciparum* transmission only: Cape Verde [49], the Dominican Republic [50], Haiti [50], [51] and Mayotte [52]. This resulted in a global total of 95 *Pv*MECs. Figure 1 summarises the various layers applied on the 95 *Pv*MECs in order to derive the limits of *P. vivax* transmission. The results of these different steps are described below.

### Defining the spatial limits of *P. vivax* transmission at sub-national level

**Figure 2:**
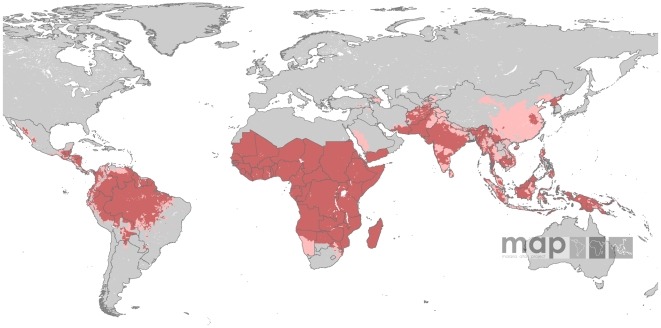
*Plasmodium vivax* malaria risk defined by *Pv*API data. Transmission was defined as stable (red areas, where *Pv*API≥0.1 per 1,000 people p.a.), unstable (pink areas, where *Pv*API<0.1 per 1,000 p.a.) or no risk (grey areas). The boundaries of the 95 countries defined as *P. vivax* endemic are shown.

*Pv*API data were available for 51 countries. Data for four countries were available up to 2009. For 29 countries the last year of reporting was 2008, whilst 2007 and 2006 were the last years available for 11 and six countries, respectively. For Colombia the last reporting year was 2005. No HMIS data could be obtained for Kyrgyzstan and Uzbekistan, for which information contained in the most recent travel and health guidelines [25], [26] was used to map risk. With the exception of Namibia, Saudi Arabia, South Africa and Swaziland, which were treated like all other nations, no HMIS data were solicited for countries in the Africa+ region, where stable risk of *P. vivax* transmission was assumed to be present throughout the country territories. In Botswana, stable risk was assumed in northern areas as specified by travel and health guidelines [25], [26]. Amongst those countries for which HMIS data were available, 16 reported at ADMIN1 and 29 at ADMIN2 level. For Southern China, Myanmar, Nepal and Peru, data were available at ADMIN3 level. Data for Namibia and Venezuela were resolved at ADMIN1 and ADMIN2 levels. In total, 17,591 administrative units were populated with *Pv*API data. Protocol S1 describes these data in detail. Figure 2 shows the spatial extent of *P. vivax* transmission as defined by the *Pv*API data, with areas categorised as malaria free, unstable (*Pv*API<0.1 case per 1,000 p.a.) or stable (*Pv*API≥0.1 case per 1,000 p.a.) transmission [29].

### Biological masks to refine the limits of transmission

**Figure 3:**
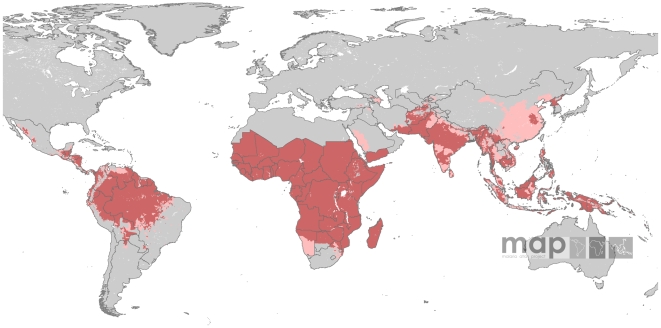
Further refinement of *Plasmodium vivax* transmission risk areas using the temperature layer of exclusion. Risk areas are defined as in Figure 2.

Figure 3 shows the limits of *P. vivax* transmission after overlaying the temperature mask on the *Pv*API surface. The *P. vivax*-specific temperature mask was less exclusive of areas of risk than that derived for *P. falciparum* [18]. Exclusion of risk was mainly evident in the Andes, the southern fringes of the Himalayas, the eastern fringe of the Tibetan plateaux, the central mountain ridge of New Guinea and the East African, Malagasy and Afghan highlands. There was a remarkable correspondence between *Pv*API defined risk in the Andean and Himalayan regions and the temperature mask, which trimmed pixels of no risk at very high spatial resolution in these areas.

**Figure 4:**
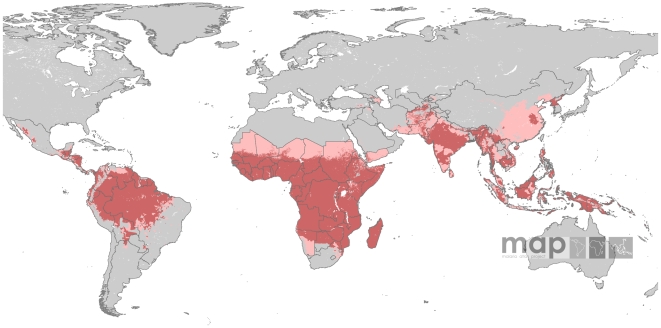
Aridity layer overlaid on the *Pv*API and temperature layers. Risk areas are defined as in Figure 2.

The aridity mask used here [36] was more contemporary and derived from higher spatial resolution imagery than the one used to define the limits of *P. falciparum* [18]. Figure 4 shows that the effects of the aridity mask were more evident in the Sahel and southern Saharan regions, as well as the Arabian Peninsula. In the western coast of Saudi Arabia, unstable risk defined by the *Pv*API layer was reduced to isolated foci of unstable risk by the aridity mask. In Yemen, stable risk was constrained to the west coast and to limited pockets along the southern coast. Similarly, endemic areas of stable risk defined by *Pv*API data in southern Afghanistan, southern Iran and throughout Pakistan were largely reduced to unstable risk by the aridity mask.

### Medical intelligence used to refine risk

The two international travel and health guidelines consulted [25], [26] cite 59 specific urban areas in 31 countries as being malaria free, in addition to urban areas in China, Indonesia (those found in Sumatra, Kalimantan, Nusa Tenggara Barat and Sulawesi) and the Philippines (Protocol S3). A total of 42 of these cities fell within areas classified as malarious and amongst these, eight were found within the range of *An. stephensi*, as were some urban areas in south-western Yunnan, China. Risk in the latter was down-regulated from stable to unstable and from unstable to free due to the presence of this urban vector. In the remaining 34 cities and other urban areas in China, Indonesia and the Philippines, risk was excluded. In addition, 36 administrative units, including islands, are cited as being malaria free (Protocol S3). These territories were excluded as areas of risk, if not already classified as such by the *Pv*API surface and biological masks. In addition, the island of Aneityum, in Vanuatu [53], the area around Angkor Watt, in Cambodia, and the island of Socotra, in Yemen [54], were classified as malaria free following additional medical intelligence and personal communication with malaria experts from these countries.

### Frequency of Duffy negativity

From the assembled library of references, 821 spatially unique Duffy blood type surveys were identified. Globally the data points were spatially representative, with 265 in America, 213 in Africa+ (167 sub-Saharan), 207 in CSE Asia and 136 in Europe. The total global sampled population was 131,187 individuals, with 24,816 (18.9%) in Africa+ and 33 African countries represented in the final database.

**Figure 5:**
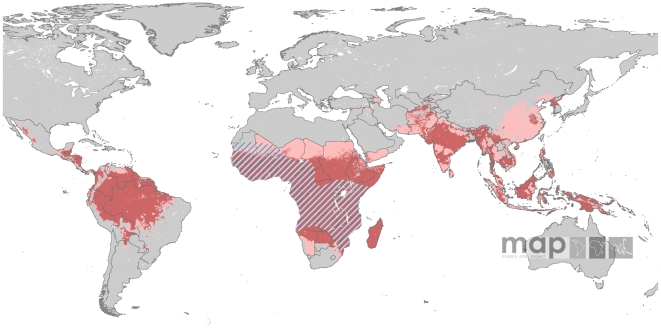
The global spatial limits of *Plasmodium vivax* malaria transmission in 2009. Risk areas are defined as in Figure 2. The medical intelligence and predicted Duffy negativity layers are overlaid on the *P. vivax* limits of transmission as defined by the *Pv*API data and biological mask layers. Areas where Duffy negativity prevalence was estimated as ≥90% are hatched, indicating where PAR estimates were modulated most significantly by the presence of this genetic trait.

The modelled global map of Duffy negativity (Figure 5) indicates that the *P. vivax* resistant phenotype is rarely seen outside of Africa, and, when this is the case, it is mainly in localised New World migrant communities. Within Africa, the predicted prevalence was strikingly high south of the Sahara. Across this region, the silent Duffy allele was close to fixation in 31 countries with 95% or more of the population being Duffy negative. Frequencies fell sharply into southern Africa and into the Horn of Africa. For instance, the frequency of Duffy negativity in the South African population was 62.7%, increasing to 65.0% in Namibia and 73.5% across Madagascar. The situation was predicted to be highly heterogeneous across Ethiopia, with an estimated 50.0% of the overall population being Duffy negative.

### Populations at risk of *P. vivax* transmission

The estimated *P. vivax* endemic areas and PAR for 2009 are presented in Table 1, stratified by unstable (*Pv*API<0.1 per 1,000 p.a.) and stable (*Pv*API≥0.1 per 1,000 p.a.) risk of transmission, globally and by region and sub-region. It was estimated that there were 2.85 billion people at risk of *P. vivax* transmission worldwide in 2009, the vast majority (91.0%) inhabiting the CSE Asia region, 5.5% living in America and 3.4% living in Africa+, after accounting for Duffy negativity. An estimated 57.1% of the *P. vivax* PAR in 2009 lived in areas of unstable transmission, with a population of 1.63 billion.

**Table 1:** Regional and global areas and PAR of *Plasmodium vivax* malaria in 2009.

Region	Area (km^2^)			PAR (millions)		
	Unstable	Stable	Any risk	Unstable	Stable	Any risk
Africa+	4,812,618	17,980,708	22,793,326	20.1	77.9	98.0
America	1,368,380	8,087,335	9,455,715	99.0	58.8	157.8
CSE Asia	5,848,939	6,127,549	11,976,488	1,509.0	1,084.2	2,593.2
*West Asia*	*2,007,247*	*2,800,612*	*4,807,859*	*653.9*	*845.2*	*1,499.2*
*Central Asia*	*3,156,574*	*1,277,219*	*4,433,793*	*694.3*	*129.2*	*823.4*
*East Asia*	*685,118*	*2,049,717*	*2,734,835*	*160.8*	*109.8*	*270.6*
World	12,029,937	32,195,600	44,225,537	1,628.1	1,220.9	2,849.0

Country level PAR estimates are provided in Protocol S4. The ten countries with the highest estimated PAR, in descending order, were: India, China, Indonesia, Pakistan, Viet Nam, Philippines, Brazil, Myanmar, Thailand and Ethiopia. PAR estimates in India accounted for 41.9% of the global PAR estimates, with 60.3% of the more than one billion PAR (1.19 billion) living in stable transmission areas. The situation in China was different as, according to the *Pv*API input data, areas of stable transmission were only found in the southern provinces of Yunnan and Hainan, and in the north-eastern province of Anhui, which reported an unusually high number of cases up to 2007. The latter is in accordance with a recent report documenting the resurgence of malaria in this province [55]. Transmission in the rest of China was largely negligible, with *Pv*API values well below 0.1 case per 1,000 people p.a. Given the reported cases, however, these were classified as unstable transmission areas and the total PAR estimated within them, after urban exclusions, was 583 million people. All other countries reporting the highest PAR were in CSE Asia, with the exception of Brazil and Ethiopia.

## Discussion

We present a contemporary evidence-based map of the global distribution of *P. vivax* transmission developed from a combination of mapped sub-national HMIS data, biological rules of transmission exclusion and medical intelligence. The methods used were developed from those implemented for *P. falciparum* malaria [18] and can be reproduced following the sequence of data layer assemblies and exclusions illustrated in Figure 1.

*Plasmodium vivax* is transmitted within 95 countries in tropical, sub-tropical and temperate regions, reaching approximately 43 degrees north in China and approximately 30 degrees south in Southern Africa. The fact that *P. vivax* has a wider range than *P. falciparum* [18] is facilitated by two aspects of the parasite's biology [56]: i) its development at lower temperatures during sporogony [31]; and ii) its ability to produce hypnozoites during its life cycle in the human host [57]. The sporogonic cycle of *P. vivax* is shorter (i.e. a lower number of degree days required for its completion) and the parasite's sexual stage is active at lower temperatures than other human malaria parasites (Protocol S2) [31]. Consequently, generation of sporozoites is possible at higher altitudes and more extreme latitudes. In the human host, hypnozoites of *P. vivax* temperate strains can relapse anywhere between months and years after the initial infection, often temporally coincident with optimal climatic conditions in a new transmission season [10], [57].

The resulting maps produced an estimate of 2.85 billion people living at risk of *P. vivax* malaria transmission in 2009. The distribution of *P. vivax* PAR is very different from that of *P. falciparum* [18], due to the widespread distribution of *P. vivax* in Asia, up to northern China, and the high prevalence of the Duffy negativity phenotype in Africa. China accounts for 22.0% of the global estimated *P. vivax* PAR, although 93.1% of these people live in areas defined as unstable transmission (Protocol S4). An important caveat is that *Pv*API data from central and northern China could only be accessed at the lowest administrative level (ADMIN1) (Protocol S1). The very high population densities found in this country exacerbate the problem, inevitably biasing PAR estimates, despite urban areas in China being excluded from the calculations following information from the sources of medical intelligence that were consulted [25], [26]. Malaria transmission in most of these unstable transmission areas in China is probably negligible given the very few cases reported between 2003 and 2007. It is important to stress the necessity to access *Pv*API data at a higher spatial resolution from China (i.e. at the county level) in order to refine these estimates and minimise biases.

In Africa, the modelled prevalence of Duffy negativity shows that very high rates of this phenotype are present in large swaths of West and Central Africa (Figure 5). One of the functions of the Duffy antigen is being a receptor of *P. vivax* [46] and its absence has been shown to preclude infection with this parasite [58], [59], although the extent of this has been questioned [60]–[63]. There is no doubt that the African continent has a climate highly conducive to *P. vivax* transmission (Protocol S2). Moreover, dominant African *Anopheles* have been shown to be competent vectors of this parasite [62], [64], [65]. In addition, there is a plethora of evidence of *P. vivax* transmission in Africa, mostly arising from travel-acquired *P. vivax* infections during visits to malaria endemic African countries (Table 2; Protocol S1). This evidence supports the hypothesis that *P. vivax* may have been often misdiagnosed as *P. ovale* in the region due to a combination of morphological similarity and the prevailing bio-geographical dogma driven by the high prevalence of Duffy negativity [60]. Despite the fact that the risk of *P. vivax* is cosmopolitan, PAR estimates in Africa were modulated according to the high limitations placed on infection by the occurrence of the Duffy negative trait. Consequently, the PAR in the Africa+ region accounts for only 3.5% of the global estimated *P. vivax* PAR. Although recent work has shown 42 *P. vivax* infections amongst 476 individuals genotyped as Duffy negative across eight sites in Madagascar [63], we have taken a conservative approach and consider it premature to relax the Duffy exclusion of PAR across continental Africa until this study has been replicated elsewhere.

**Table 2:** Published evidence of *Plasmodium vivax* malaria transmission in African countries.

Country	References*
Angola	[68]–[73]
Benin	[68], [70], [71], [74]
Botswana	[72]
Burkina Faso	[68], [71]
Burundi	[70]–[73]
Cameroon	[68], [69], [71]–[79]
Cen. African Rep.	[68]
Chad	[74]
Comoros	[68]
Congo	[68], [70], [71], [73], [74], [76], [77], [80]
Côte d'Ivoire	[68]–[71], [73], [74], [76], [78]
Congo (DR)	[68], [81]
Djibouti	[68], [78]
Equatorial Guinea	[82]
Eritrea	[71], [73], [76], [77], [83], [84]
Ethiopia	[68]–[74], [76]–[79], [85]
Gabon	[68], [71], [86]
Gambia	[71], [72], [76], [78]
Ghana	[69]–[74], [76]–[79]
Guinea	[68], [69], [71], [76], [77]
Kenya	[68]–[73], [76]–[79]
Liberia	[68]–[73], [76]–[79]
Madagascar	[68]–[73], [76], [78], [87]
Malawi	[68], [70], [72], [73]
Mali	[68], [69], [71]
Mauritania	[68], [69], [71], [72], [76], [77], [88], [89]
Mozambique	[68]–[71], [73], [76], [79], [90]
Namibia	[70]
Niger	[68], [69], [71], [76]
Nigeria	[69]–[74], [76]–[79], [91]
Rwanda	[68], [71], [72], [78]
São Tomé and Príncipe	[68], [92]
Senegal	[68], [70], [71], [73], [76], [77]
Sierra Leone	[68], [69], [72]–[74], [76], [78]
Somalia	[69], [70], [78], [79], [93]
South Africa	[69]–[71], [76]–[78]
Sudan	[68]–[74], [76], [77], [79], [94]
Togo	[70], [71]
Uganda	[69]–[74], [76]–[79], [95]
Tanzania	[68]–[72], [76], [77], [79]
Zambia	[69]–[72], [78], [96]
Zimbabwe	[68], [69], [71]

*The cited references mostly document imported cases from Africa. Evidence of transmission of *P. vivax* in Guinea Bissau and Swaziland could not be found in the published literature.

Mapping the distribution of *P. vivax* malaria has presented a number of unique challenges compared to *P. falciparum*, some of which have been addressed by the methods used here. The influence of climate on parasite development has been allowed for by implementing a temperature mask parameterised specifically for the *P. vivax* life cycle. The question of Duffy negativity and *P. vivax* transmission has also been addressed by modelling the distribution of this phenotype and by allowing the predicted prevalence to modulate PAR. It is also worth noting that the accuracy of HMIS for *P. vivax* clinical cases, particularly in areas of coincidental *P. falciparum* risk, is notoriously poor [66], in part because microscopists are less likely to record the presence of a parasite assumed to be clinically less important. Here, HMIS data were averaged over a period of up to four years and used to differentiate malaria free areas from those that are malarious. Within the latter, a conservative threshold was applied to classify risk areas as being of unstable (*Pv*API<0.1 per 1,000 p.a.) or stable (*Pv*API≥0.1 per 1,000 p.a.) transmission [29]. We believe that this conservative use of HMIS data balances, to some extent, anomalies introduced by *P. vivax* underreporting and the correspondence of the biological masks and *Pv*API data in many areas is reassuring.

The intensity of transmission within the defined stable limits of *P. vivax* risk will vary across this range and this will be modelled using geostatistical techniques similar to those developed recently for *P. falciparum* [19]. This modelling work will be cognisant of the unique epidemiology of *P. vivax*. First, in areas where *P. vivax* infection is coincidental with *P. falciparum*, prevalence of the former may be suppressed by cross-species immunity [67] or underestimated by poor diagnostics [66]. Second, there is the ability of *P. vivax* to generate hypnozoites that lead to relapses. These characteristics render the interpretation of prevalence measures more problematic [5]. Third, the prevalence of Duffy negativity provides protection against infection in large sections of the population in Africa [58], [59]. An appropriate modelling framework is under development and will be the subject of a subsequent paper mapping *P. vivax* malaria endemicity using parasite prevalence data. These data are being collated in the MAP database, with nearly 9,000 *P. vivax* parasite rate records archived by 01 March 2010.

## Supporting Information

Protocol S1 Defining risk of transmission of *Plasmodium vivax* using case reporting data. Document describing more extensively one of the layers used to create the final map.
(2.87 MB DOC)

Protocol S2 Defining the global biological limits of *Plasmodium vivax* transmission. Document describing more extensively two of the layers used to create the final map.
(0.42 MB DOC)

Protocol S3 Risk modulation based upon medical intelligence. Document describing more extensively one of the layers used to create the final map.
(0.36 MB DOC)

Protocol S4 Country level area and population at risk of *Plasmodium vivax* malaria in 2009. Country-level table of the estimated area and populations at risk of *P. vivax* malaria in 2009
(0.16 MB DOC)

## References

[1] Snow RW, Craig MH, Newton CRJC, Steketee RW (2003). The public health burden of *Plasmodium falciparum* malaria in Africa: deriving the numbers. Working Paper No. 11.

[2] Hay SI, Guerra CA, Tatem A, Atkinson P, Snow RW (2005). Urbanization, malaria transmission and disease burden in Africa.. Nat Rev Microbiol.

[3] Mendis K, Sina BJ, Marchesini P, Carter R (2001). The neglected burden of *Plasmodium vivax* malaria.. Am J Trop Med Hyg.

[4] Baird JK (2007). Neglect of *Plasmodium vivax* malaria.. Trends Parasitol.

[5] Price RN, Tjitra E, Guerra CA, Yeung S, White NJ (2007). Vivax malaria: neglected and not benign.. Am J Trop Med Hyg.

[6] Mueller I, Galinski MR, Baird JK, Carlton JM, Kochar DK (2009). Key gaps in the knowledge of *Plasmodium vivax*, a neglected human malaria parasite.. Lancet Infect Dis.

[7] Guerra CA, Snow RW, Hay SI (2006). Defining the global spatial limits of malaria transmission in 2005.. Adv Parasitol.

[8] Guerra CA, Snow RW, Hay SI (2006). Mapping the global extent of malaria in 2005.. Trends Parasitol.

[9] Sattabongkot J, Tsuboi T, Zollner GE, Sirichaisinthop J, Cui L (2004). *Plasmodium vivax* transmission: chances for control?. Trends Parasitol.

[10] Baird JK (2009). Resistance to therapies for infection by *Plasmodium vivax*.. Clin Microbiol Rev.

[11] Genton B, D'Acremont V, Rare L, Baea K, Reeder JC (2008). *Plasmodium vivax* and mixed infections are associated with severe malaria in children: a prospective cohort study from Papua New Guinea.. PLoS Med.

[12] Tjitra E, Anstey NM, Sugiarto P, Warikar N, Kenangalem E (2008). Multidrug-resistant *Plasmodium vivax* associated with severe and fatal malaria: a prospective study in Papua, Indonesia.. PLoS Med.

[13] Anstey NM, Russell B, Yeo TW, Price RN (2009). The pathophysiology of vivax malaria.. Trends Parasitol.

[14] Kochar DK, Das A, Kochar SK, Saxena V, Sirohi P (2009). Severe *Plasmodium vivax* malaria: a report on serial cases from Bikaner in northwestern India.. Am J Trop Med Hyg.

[15] Barcus MJ, Basri H, Picarima H, Manyakori C, Sekartuti (2007). Demographic risk factors for severe and fatal vivax and falciparum malaria among hospital admissions in northeastern Indonesian Papua.. Am J Trop Med Hyg.

[16] Parakh A, Agarwal N, Aggarwal A, Aneja A (2009). *Plasmodium vivax* malaria in children: uncommon manifestations.. Ann Trop Paediatr.

[17] Hay SI, Snow RW (2006). The Malaria Atlas Project: Developing global maps of malaria risk.. PLoS Med.

[18] Guerra CA, Gikandi PW, Tatem AJ, Noor AM, Smith DL (2008). The limits and intensity of *Plasmodium falciparum* transmission: implications for malaria control and elimination worldwide.. PLoS Med.

[19] Hay SI, Guerra CA, Gething PW, Patil AP, Tatem AJ (2009). A world malaria map: *Plasmodium falciparum* endemicity in 2007.. PLoS Med.

[20] Snow RW, Guerra CA, Mutheu JJ, Hay SI (2008). International funding for malaria control in relation to populations at risk of stable *Plasmodium falciparum* transmission.. PLoS Med.

[21] Gething PW, Patil AP, Hay SI (2010). Quantifying aggregated uncertainty in *Plasmodium falciparum* malaria prevalence and populations at risk via efficient space-time geostatistical joint simulation.. PLoS Comput Biol.

[22] Hay SI, Okiro EA, Gething PW, Patil AP, Tatem AJ (2010). Estimating the global clinical burden of *Plasmodium falciparum* malaria in 2007.. PLoS Med.

[23] Feachem R, Sabot O (2008). A new global malaria eradication strategy.. Lancet.

[24] Roll Back Malaria Partnership (2008). The Global Malaria Action Plan: For a malaria-free world..

[25] Centers for Disease Control and Prevention (2009). CDC Health Information for International Travel 2010.

[26] WHO (2010). International Travel and Health: Situation as on 1 January 2010.

[27] FAO (2008). The Global Administrative Unit Layers (GAUL): Technical Aspects.

[28] Pampana E (1969). A textbook of malaria eradication.

[29] Hay SI, Smith DL, Snow RW (2008). Measuring malaria endemicity from intense to interrupted transmission.. Lancet Infect Dis.

[30] Yekutiel P, Klingberg MA (1980). III The Global Malaria Eradication Campaign.. Eradication of infectious diseases: a critical study.

[31] Nikolaev BP (1935). On the influence of temperature on the development of malaria *plasmodia* inside the mosquito.. Leningrad Pasteur Institute of Epidemiology and Bacteriology.

[32] Hijmans RJ, Cameron SE, Parra JL, Jones PG, Jarvis A (2005). Very high resolution interpolated climate surfaces for global land areas.. Int J Climatol.

[33] Kiszewski A, Mellinger A, Spielman A, Malaney P, Sachs SE (2004). A global index representing the stability of malaria transmission.. Am J Trop Med Hyg.

[34] Shililu JI, Grueber WB, Mbogo CM, Githure JI, Riddiford LM (2004). Development and survival of *Anopheles gambiae* eggs in drying soil: influence of the rate of drying, egg age, and soil type.. J Am Mosq Control Assoc.

[35] Gray EM, Bradley TJ (2005). Physiology of desiccation resistance in *Anopheles gambiae* and *Anopheles arabiensis*.. Am J Trop Med Hyg.

[36] Bicheron P, Defourny P, Brockmann C, Schouten L, Vancutsem C (2008). GLOBCOVER: Products Description and Validation Report.

[37] Omer SM, Cloudsley-Thompson JL (1970). Survival of female *Anopheles gambiae* Giles through a 9-month dry season in Sudan.. Bull World Health Organ.

[38] Omer SM, Cloudsley-Thomson JL (1968). Dry season biology of *Anopheles gambiae* Giles in the Sudan.. Nature.

[39] Bouma MJ, Parvez SD, Nesbit R, Winkler AM (1996). Malaria control using permethrin applied to tents of nomadic Afghan refugees in northern Pakistan.. Bull World Health Organ.

[40] Balk DL, Deichmann U, Yetman G, Pozzi F, Hay SI (2006). Determining global population distribution: methods, applications and data.. Adv Parasitol.

[41] Hay SI, Sinka ME, Okara RM, Kabaria CW, Mbithi PM (2010). Developing maps of the dominant *Anopheles* vectors of human malaria.. PLoS Med.

[42] Miller LH, Mason SJ, Clyde DF, McGinniss MH (1976). The resistance factor to *Plasmodium vivax* in blacks. The Duffy-blood-group genotype, FyFy.. N Engl J Med.

[43] Mourant AE, Kopec AC, Domaniewska-Sobczak K (1976). The Distribution of the Human Blood Groups and other Polymorphisms.

[44] Cavalli-Sforza LL, Menozzi P, Piazza A (1994). The History and Geography of Human Genes.

[45] Cutbush M, Mollison PL (1950). The Duffy blood group system.. Heredity.

[46] Langhi DM, Bordin JO (2006). Duffy blood group and malaria.. Hematology.

[47] UNPD (2008). World Urbanization Prospects: The 2007 Revision Population Database.. http://esa.un.org/unpp/.

[48] Hay SI, Noor AM, Nelson A, Tatem AJ (2005). The accuracy of human population maps for public health application.. Trop Med Int Health.

[49] Ministério da Saúde de Cabo VerdeDirecção Geral de SaúdePrograma Nacional de Luta Contra o Paludismo (2009). Plano Estratégico de Pré-Eliminação do Paludismo 2009–2013..

[50] PAHO (2006). Regional Strategic Plan for Malaria in the Americas 2006–2010.

[51] Lindo JF, Bryce JH, Ducasse MB, Howitt C, Barrett DM (2007). *Plasmodium malariae* in Haitian refugees, Jamaica.. Emerg Infect Dis.

[52] Tchen J, Ouledi A, Lepere JF, Ferrandiz D, Yvin JL (2006). Epidemiologie et prevention du paludisme dans les iles du sud-ouest de l'Ocean Indien.. Med Trop (Mars).

[53] Kaneko A, Taleo G, Kalkoa M, Yamar S, Kobayakawa T (2000). Malaria eradication on islands.. Lancet.

[54] EMRO (2008). Technical discussion on malaria elimination in the Eastern Mediterranean Region: vision, requirements and strategic outline..

[55] Zhang W, Wang L, Fang L, Ma J, Xu Y (2008). Spatial analysis of malaria in Anhui province, China.. Malar J.

[56] Coatney GR, Collins WE, Warren M, Contacos PG (2003). The primate malarias.[CD-ROM; original book published 1971].

[57] Garnham PCC, Wernsdorfer WH, McGregor I (1988). Malaria parasites of man: life-cycles and morphology (excluding ultrastructure).. Malaria: principles and practice of malariology.

[58] Welch SG, McGregor IA, Williams K (1977). The Duffy blood group and malaria prevalence in Gambian West Africans.. Trans R Soc Trop Med Hyg.

[59] Mathews HM, Armstrong JC (1981). Duffy blood types and vivax malaria in Ethiopia.. Am J Trop Med Hyg.

[60] Rosenberg R (2007). *Plasmodium vivax* in Africa: hidden in plain sight?. Trends Parasitol.

[61] Cavasini CE, Mattos LC, Couto AA, Bonini-Domingos CR, Valencia SH (2007). *Plasmodium vivax* infection among Duffy antigen-negative individuals from the Brazilian Amazon region: an exception?. Trans R Soc Trop Med Hyg.

[62] Ryan JR, Stoute JA, Amon J, Dunton RF, Mtalib R (2006). Evidence for transmission of *Plasmodium vivax* among a duffy antigen negative population in Western Kenya.. Am J Trop Med Hyg.

[63] Menard D, Barnadas C, Bouchier C, Henry-Halldin C, Gray LR (2010). *Plasmodium vivax* clinical malaria is commonly observed in Duffy-negative Malagasy people.. Proc Natl Acad Sci U S A.

[64] Collins WE, Roberts JM (1991). *Anopheles gambiae* as a host for geographic isolates of *Plasmodium vivax*.. J Am Mosq Control Assoc.

[65] Taye A, Hadis M, Adugna N, Tilahun D, Wirtz RA (2006). Biting behavior and *Plasmodium* infection rates of *Anopheles arabiensis* from Sille, Ethiopia.. Acta Trop.

[66] Mayxay M, Pukrittayakamee S, Newton PN, White NJ (2004). Mixed-species malaria infections in humans.. Trends Parasitol.

[67] Maitland K, Williams TN, Newbold CI (1997). *Plasmodium vivax* and *P. falciparum*: Biological interactions and the possibility of cross-species immunity.. Parasitol Today.

[68] Gautret P, Legros F, Koulmann P, Rodier MH, Jacquemin JL (2001). Imported *Plasmodium vivax* malaria in France: geographical origin and report of an atypical case acquired in Central or Western Africa.. Acta Trop.

[69] Holtz TH, Kachur SP, MacArthur JR, Roberts JM, Barber AM (2001). Malaria surveillance–United States, 1998.. MMWR CDC Surveill Summ.

[70] Newman RD, Barber AM, Roberts J, Holtz T, Steketee RW (2002). Malaria surveillance–United States, 1999.. MMWR Surveill Summ.

[71] Causer LM, Newman RD, Barber AM, Roberts JM, Stennies G (2002). Malaria surveillance–United States, 2000.. MMWR Surveill Summ.

[72] Filler S, Causer LM, Newman RD, Barber AM, Roberts JM (2003). Malaria surveillance–United States, 2001.. MMWR Surveill Summ.

[73] Thwing J, Skarbinski J, Newman RD, Barber AM, Mali S (2007). Malaria surveillance–United States, 2005.. MMWR Surveill Summ.

[74] Mali S, Steele S, Slutsker L, Arguin PM (2008). Malaria surveillance–United States, 2006.. MMWR Surveill Summ.

[75] Durante Mangoni E, Severini C, Menegon M, Romi R, Ruggiero G (2003). Case report: An unusual late relapse of *Plasmodium vivax* malaria.. Am J Trop Med Hyg.

[76] Shah S, Filler S, Causer LM, Rowe AK, Bloland PB (2004). Malaria surveillance–United States, 2002.. MMWR Surveill Summ.

[77] Eliades MJ, Shah S, Nguyen-Dinh P, Newman RD, Barber AM (2005). Malaria surveillance–United States, 2003.. MMWR Surveill Summ.

[78] Skarbinski J, James EM, Causer LM, Barber AM, Mali S (2006). Malaria surveillance–United States, 2004.. MMWR Surveill Summ.

[79] Mali S, Steele S, Slutsker L, Arguin PM (2009). Malaria surveillance–United States, 2007.. MMWR Surveill Summ.

[80] Culleton R, Ndounga M, Zeyrek FY, Coban C, Casimiro PN (2009). Evidence for the transmission of *Plasmodium vivax* in the Republic of the Congo, West Central Africa.. J Infect Dis.

[81] Comellini L, Tozzola A, Baldi F, Brusa S, Serra L (1998). *Plasmodium vivax* congenital malaria in a newborn of a Zairian immigrant.. Ann Trop Paediatr.

[82] Rubio JM, Benito A, Roche J, Berzosa PJ, Garcia ML (1999). Semi-nested, multiplex polymerase chain reaction for detection of human malaria parasites and evidence of *Plasmodium vivax* infection in Equatorial Guinea.. Am J Trop Med Hyg.

[83] Peruzzi S, Gorrini C, Piccolo G, Calderaro A, Dettori G (2007). Prevalence of imported malaria in Parma during 2005–2006.. Acta Biomed.

[84] Sintasath DM, Ghebremeskel T, Lynch M, Kleinau E, Bretas G (2005). Malaria prevalence and associated risk factors in Eritrea.. Am J Trop Med Hyg.

[85] Teka H, Petros B, Yamuah L, Tesfaye G, Elhassan I (2008). Chloroquine-resistant *Plasmodium vivax* malaria in Debre Zeit, Ethiopia.. Malar J.

[86] Poirriez J, Landau I, Verhaeghe A, Savage A, Dei-Cas E (1991). [Atypical forms of *Plasmodium vivax*. Apropos of a case].. Ann Parasitol Hum Comp.

[87] Rabarijaona LP, Randrianarivelojosia M, Raharimalala LA, Ratsimbasoa A, Randriamanantena A (2009). Longitudinal survey of malaria morbidity over 10 years in Saharevo (Madagascar): further lessons for strengthening malaria control.. Malar J.

[88] Cortes H, Morillas-Marquez F, Valero A (2003). Malaria in Mauritania: the first cases of malaria endemic to Nouakchott.. Trop Med Int Health.

[89] Lekweiry KM, Abdallahi MO, Ba H, Arnathau C, Durand P (2009). Preliminary study of malaria incidence in Nouakchott, Mauritania.. Malar J.

[90] Wejda BU, Huchzermeyer H, Dormann AJ (2002). Hotel malaria in Greece: Mozambique origin, American vector, German victims.. J Travel Med.

[91] Erhabor O, Babatunde S, Uko KE (2006). Some haematological parameters in plasmodial parasitized HIV-infected Nigerians.. Niger J Med.

[92] Snounou G, Pinheiro L, Antunes AM, Ferreira C, do Rosario VE (1998). Non-immune patients in the Democratic Republic of Sao Tome e Principe reveal a high level of transmission of *P. ovale* and *P. vivax* despite low frequency in immune patients.. Acta Trop.

[93] Peragallo MS, Sabatinelli G, Majori G, Cali G, Sarnicola G (1997). Prevention and morbidity of malaria in non-immune subjects; a case-control study among Italian troops in Somalia and Mozambique, 1992–1994.. Trans R Soc Trop Med Hyg.

[94] Himeidan YE, Elbashir MI, El-Rayah el A, Adam I (2005). Epidemiology of malaria in New Halfa, an irrigated area in eastern Sudan.. East Mediterr Health J.

[95] Illamperuma C, Allen BL (2007). Pulmonary edema due to *Plasmodium vivax* malaria in an American missionary.. Infection.

[96] Blossom DB, King CH, Armitage KB (2005). Occult *Plasmodium vivax* infection diagnosed by a polymerase chain reaction-based detection system: a case report.. Am J Trop Med Hyg.

