# Supplementary material for: The International Limits and Population at Risk of Plasmodium vivax Transmission in 2009
Source: PLoS Negl Trop Dis. 2010 Aug 3;4(8):e774. doi: 10.1371/journal.pntd.0000774 (PMC2914753; doi:10.1371/journal.pntd.0000774)
Supplement: Protocol S1 — Defining risk of transmission of Plasmodium vivax using case reporting data. Document describing more extensively one of the layers used to create the final map. (2.87 MB DOC) [file pntd.0000774.s001.doc]

**PROTOCOL S1: Defining risk of transmission of *Plasmodium vivax* using case reporting data**

**Identification of *Plasmodium vivax* malaria endemic countries**

In order to define *Plasmodium vivax* malaria endemic countries (*Pv*MECs), two sets of international travel and health guidelines [1,2] were used to list all countries potentially supporting some level of *P. vivax* transmission. This yielded 109 countries in the three regions: America (22 countries), the Africa+ region (African countries plus Yemen and Saudi Arabia, due to the presence of *Anopheles arabiensis*) (52 countries) and Central and South East (CSE) Asia (35 countries).

Nineteen *Pv*MECs were identified in America. All countries in Mesoamerica and South America are *P. vivax* endemic, except for Uruguay and Chile. There is no *P. vivax* transmission in the Caribbean, where the only two malaria endemic countries, Dominican Republic and Haiti, report *P. falciparum* transmission only [3,4]. The very limited risk found in Jamaica after an outbreak in 2006/7 [5] was also exclusively due to *P. falciparum*.

All 22 countries with *P. falciparum* malaria endemicity in CSE Asia are also *P. vivax* endemic. Eight countries towards the more temperate zones of Eastern Europe and Central Asia report only *P. vivax* transmission at present. These countries are, from West to East, Turkey, Georgia, Azerbaijan, Iraq, Uzbekistan, Kyrgyzstan, Korea Democratic People’s Republic (DPR) and the Republic of Korea. Armenia, Syrian Arab Republic and Turkmenistan have not reported locally transmitted *P. vivax* malaria cases in recent years and were thus not classified as currently *P. vivax* endemic, although none have received “certification of eradication” by the World Health Organization [6]. In Oman, four cases were reported in Manah district in 2007 and eight in Sohar district in 2008 (Al-Zedjali, pers. comm.) after the interruption, in 2003, of sporadic transmission [2]. Oman, therefore, was not classified as a *Pv*MEC. In the Russian Federation, very limited risk of *P. vivax* transmission is reported in areas of intense migration [2] and it was, therefore, not considered a *Pv*MEC.

The high prevalence of the Duffy negativity phenotype in indigenous populations of Africa, particularly of Central and West Africa, has led to the dogma that *P. vivax* is absent from large areas of the continent. Climatic conditions in most of Africa, however, are favourable to the completion of the sporogonic cycle of *P. vivax* and African anophelines have been shown to be receptive to this parasite [7]. Moreover, recent reports show that Duffy negativity does not necessarily confer complete protection against *P. vivax* infection [8-10]. Apart from the well documented transmission of *P. vivax* in countries of the Horn of Africa, published evidence confirms that *P. vivax* transmission is present in several countries of the continent, including Angola [11], Democratic Republic of the Congo [12], Equatorial Guinea [13], Madagascar [14-17], Mauritania [18], Republic of the Congo [19] and São Tomé and Príncipe [20]. Evidence also exists of *P. vivax* infections imported to non-endemic countries from many African countries. In France, 275 imported *P. vivax* cases from Africa were recorded between 1995 and 1998 [21]. The origin of these cases included countries in West Africa (n=45), Central Africa (n=28), East and South Africa (n=22) and the Indian Ocean Islands (n=180). A large retrospective analysis of *P. vivax* malaria imported to Europe showed that 11.4% and 5.5% of 618 cases originated in West and Central Africa, respectively [22]. Other reports have documented *P. vivax* importation from Equatorial Guinea [23], Mozambique [24] and Somalia [25-27]. Lastly, the latest Centers for Disease Control and Prevention (CDC) traveller’s health book features *P. vivax* amongst the malaria parasite species responsible for infections in most African countries, with frequencies ranging from rare to 15% [1].

Based on the reviewed evidence, a total of 46 countries and territories in Africa+ were classified as *Pv*MECs. Exceptions were Cape Verde and Mayotte, where only *P. falciparum* transmission is documented to exist [28,29], as well as Algeria, Egypt, Morocco and Mauritius, all of which have previously been malaria endemic but are presently classified as malaria free or with no indigenous transmission in recent years by the two sets of travel and health guidelines consulted [1,2]. Figure 1 shows the 95 countries classified as *Pv*MECs and Table 1 lists them by region and sub-region.


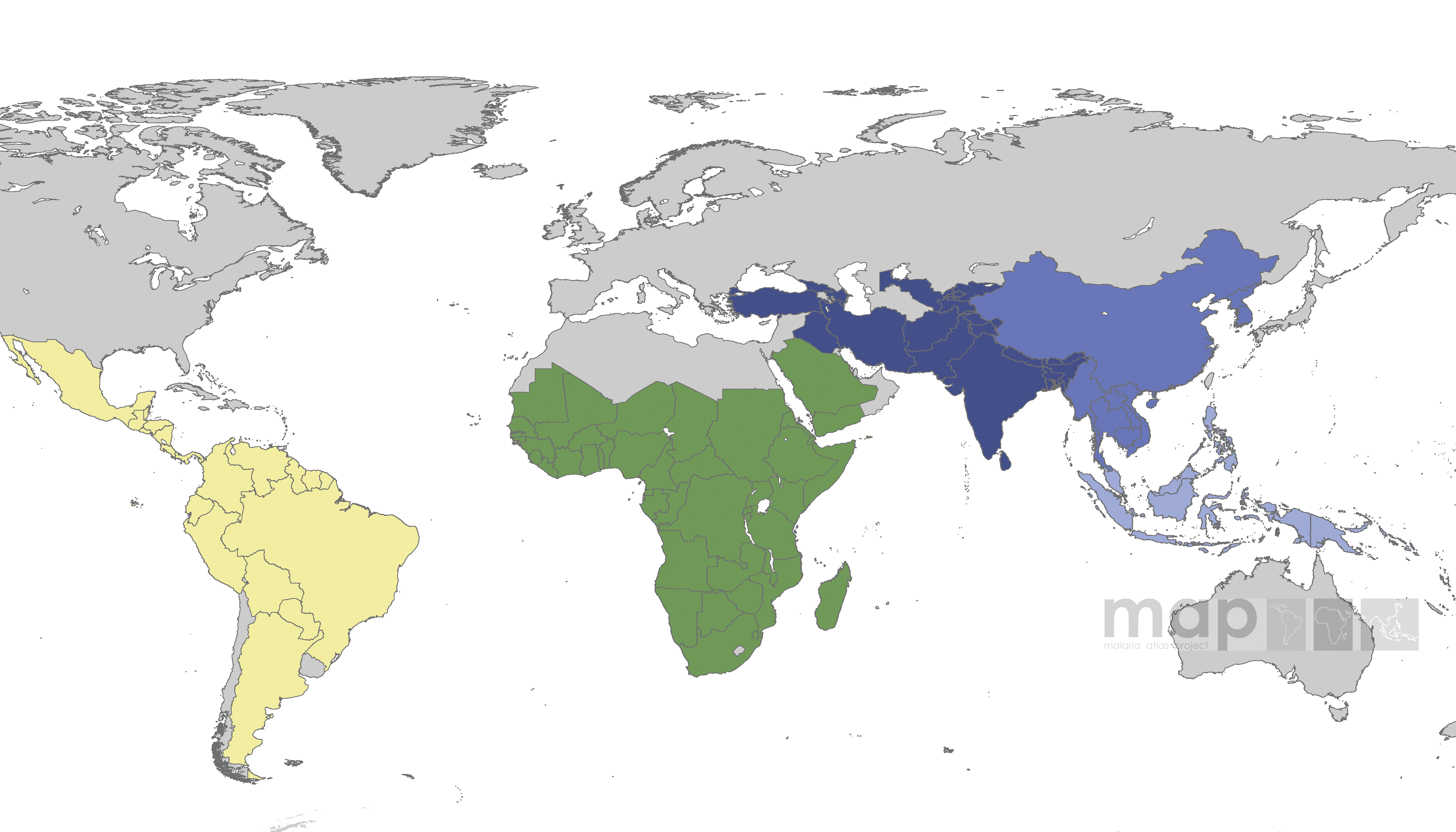


**Protocol S1, Figure 1**. *Pv*MECs by region: America (yellow), Africa+ (green) and CSE Asia (dark blue, West Asia; middle blue, Central Asia; light blue, East Asia).

**Protocol S1, Table 1**. *Pv*MECs by regions and sub-regions as per Figure 1.

| **Africa+** | **America** | **CSE Asia** |
| --- | --- | --- |
| Angola | Argentina | *West Asia* |
| Benin | Belize | Afghanistan |
| Botswana | Bolivia | Azerbaijan |
| Burkina Faso | Brazil | Bangladesh |
| Burundi | Colombia | Bhutan |
| Cameroon | Costa Rica | Georgia |
| Central African Rep. | Ecuador | India |
| Chad | El Salvador | Iran |
| Comoros | French Guiana | Iraq |
| Congo | Guatemala | Kyrgyzstan |
| Côte d'Ivoire | Guyana | Nepal |
| Congo (DR) | Honduras | Pakistan |
| Djibouti | Mexico | Sri Lanka |
| Equatorial Guinea | Nicaragua | Tajikistan |
| Eritrea | Panama | Turkey |
| Ethiopia | Paraguay | Uzbekistan |
| Gabon | Peru | *Central Asia* |
| Gambia | Suriname | Cambodia |
| Ghana | Venezuela | China |
| Guinea |  | Korea, DPR |
| Guinea-Bissau |  | Laos |
| Kenya |  | Myanmar |
| Liberia |  | Republic of Korea |
| Madagascar |  | Thailand |
| Malawi |  | Viet Nam |
| Mali |  | *East Asia* |
| Mauritania |  | Indonesia |
| Mozambique |  | Malaysia |
| Namibia |  | Papua New Guinea |
| Niger |  | Philippines |
| Nigeria |  | Solomon Islands |
| Rwanda |  | Timor-Leste |
| São Tomé and Príncipe | | Vanuatu |
| Saudi Arabia |  |  |
| Senegal |  |  |
| Sierra Leone |  |  |
| Somalia |  |  |
| South Africa |  |  |
| Sudan |  |  |
| Swaziland |  |  |
| Togo |  |  |
| Tanzania |  |  |
| Uganda |  |  |
| Yemen |  |  |
| Zambia |  |  |
| Zimbabwe |  |  |

**Classification of risk based on *P. vivax* annual parasite incidence data**

In order to classify risk areas of *P. vivax* transmission, methodologies used to map the spatial limits of *P. falciparum* transmission described previously [30] were adapted for *P. vivax*. Areas of extremely low, unstable transmission of *P. vivax* were assigned to administrative units reporting an annual parasite incidence (*Pv*API) of less than 0.1 case per 1,000 population per annum (p.a.), whilst those reporting a *Pv*API of ≥0.1 case per 1,000 population p.a. were classified as being of stable transmission. This criterion was found to be a reliable indicator for the cessation of indoor residual spraying during the consolidation phase of the Global Malaria Eradication Programme [31-33]. During this period, the limit was reduced from 0.5‰ as it became recognized that surveillance, including passive and active case detection, was often less accurate and reliable than nations thought: malaria often resumed after the cessation of spraying from 0.5‰, but rarely from 0.1‰. This more conservative categorization of malaria transmission also helps compensate for the vagaries of sub-national level case reporting [34-36].

***Pv*API data used**

Table 2 summarizes *Pv*API data characteristics for all *Pv*MECs for which these were available. API data were not available for any country in the Africa+ region, with the exception of Namibia, Saudi Arabia and South Africa. Unfortunately, case-reporting data for South Africa and Namibia did not discriminate between parasite species; it was assumed that risk of transmission of *P. vivax* and *P. falciparum* were equal across the mapped administrative units. Maps of confirmed cases by district in Swaziland for the years 2007-2009 were used to infer risk categories, assuming equal level of risk of transmission for *P. falciparum* and *P. vivax*. For Botswana, risk was constrained at the first administrative (ADMIN1) level using information contained in the travel and health guidelines consulted [1,2], assuming stable risk in malaria transmission areas. For other countries in this region, stable risk of *P. vivax* transmission was assumed to be present throughout their territories.

Case reporting data were not available either for Kyrgyzstan or Uzbekistan. In these countries, risk was defined at ADMIN1 and ADMIN2 levels, respectively, from information available in international travel and health guidelines [1,2].

In total, API data were not available for 44 identified *Pv*MECs. Most data for the other 51 countries were obtained through personal communication with individuals and institutions linked to malaria control in each country. The aim was to collate data for the four last years of reporting, ideally up to 2009. For four countries the last year of reporting available was 2009. For 29 countries, 2008 was the last year of reporting available, whilst 2007 and 2006 were the last years available for 11 and six countries, respectively. For Colombia, risk data were not available after 2005. In terms of the length of the period of reporting, one year of data was available for 13 countries, two years for four countries, three years for six countries and four years for 28 countries (Table 2).

Regarding the spatial resolution of the *Pv*API data, or the administrative level at which case reporting data were available, 16 countries reported at ADMIN1 level and 29 at ADMIN2 level. For southern China, Myanmar, Nepal and Peru, data were available at ADMIN3 level. In central and northern China data were available at ADMIN1 level. Data for Namibia and Venezuela were resolved at a mixture of ADMIN1 and ADMIN2 levels. The best average spatial resolution (ASR) was attained in Swaziland (ASR = 18) and the poorest in Saudi Arabia (ASR = 385). In total, 17,591 administrative units were populated with *Pv*API data (Table 2).

**Mapping *Pv*API data**

In order to map *Pv*API data consistently, they were reconciled to the 2009 version of the Global Administrative Unit Layers (GAUL) data set, implemented by the Food and Agriculture Organization of the United Nations (FAO) within the EC FAO Food Security for Action Programme [37].

**Protocol S1, Table 2**. Mapped *P. vivax* annual parasite incidence (*Pv*API) data for the countries for which they could be accessed. The data are grouped by the three global regions defined by Hay *et al.* [38]: Africa+, America and Central and South East (CSE) Asia. ADMIN1, 2 or 3 refers to the administrative division level (first, second or third level) at which data were available. The number of risk units refers to how many administrative units, at the level specified, were populated with actual data. Year start and Year end mark the start and end of the period for which data were available. ASR is the average spatial resolution of the mapped *Pv*API data, calculated as: sqrt(Country area / number of *Pv*API data units mapped). The lower the ASR the better, with values <100 desired for an optimal overall spatial resolution.

| **Region** | **Country** | **Administrative level** | **# risk units** | **Year start** | **Year end** | **ASR** | **Source** |
| --- | --- | --- | --- | --- | --- | --- | --- |
| Africa+ | Namibia | ADMIN1 & ADMIN2 | 30 | 2009 | 2009 | 166 | [39] |
| Africa+ | Saudi Arabia | ADMIN1 | 13 | 2005 | 2006 | 385 | [40] |
| Africa+ | South Africa | ADMIN2 | 257 | 2006 | 2009 | 69 | [41] |
| Africa+ | Swaziland | ADMIN2 | 53 | 2007 | 2009 | 18 | [42] |
| America | Argentina | ADMIN2 | 513 | 2008 | 2008 | 74 | [43] |
| America | Belize | ADMIN1 | 6 | 2006 | 2006 | 61 | [44] |
| America | Bolivia | ADMIN2 | 113 | 2008 | 2008 | 98 | [45] |
| America | Brazil | ADMIN2 | 5510 | 2004 | 2008 | 39 | [46] |
| America | Colombia* | ADMIN2 | 1087 | 2005 | 2005 | 32 | [47] |
| America | Costa Rica | ADMIN2 | 81 | 2006 | 2006 | 25 | [48] |
| America | Ecuador | ADMIN2 | 220 | 2005 | 2008 | 34 | [49] |
| America | El Salvador | ADMIN1 | 14 | 2006 | 2006 | 39 | [44] |
| America | French Guiana | ADMIN2 | 21 | 2006 | 2006 | 63 | [50] |
| America | Guatemala | ADMIN1 | 22 | 2006 | 2006 | 71 | [44] |
| America | Guyana | ADMIN1 | 10 | 2004 | 2007 | 145 | [51] |
| America | Honduras | ADMIN2 | 291 | 2005 | 2008 | 20 | [52] |
| America | Mexico | ADMIN2 | 2454 | 2005 | 2008 | 28 | [53] |
| America | Nicaragua | ADMIN1 | 17 | 2004 | 2007 | 87 | [54] |
| America | Panama | ADMIN2 | 68 | 2006 | 2007 | 33 | [55] |
| America | Paraguay | ADMIN2 | 219 | 2008 | 2008 | 43 | [56] |
| America | Peru | ADMIN3 | 1828 | 2005 | 2008 | 27 | [57] |
| America | Suriname | ADMIN1 | 10 | 2008 | 2008 | 121 | [43] |
| America | Venezuela | ADMIN1 & ADMIN2 | 30 | 2004 | 2008 | 175 | [58] |
| CSE Asia | Afghanistan | ADMIN2 | 398 | 2005 | 2008 | 40 | [40] |
| CSE Asia | Azerbaijan | ADMIN1 | 73 | 2005 | 2008 | 33 | [59] |
| CSE Asia | Bangladesh | ADMIN2 | 64 | 2007 | 2008 | 46 | [60] |
| CSE Asia | Bhutan | ADMIN1 | 20 | 2004 | 2007 | 43 | [61] |
| CSE Asia | Cambodia | ADMIN1 | 25 | 2005 | 2008 | 85 | [62] |
| CSE Asia | China* | ADMIN1 & ADMIN3 | 263 | 2003 | 2007 | 189 | [63] |
| CSE Asia | Georgia | ADMIN2 | 79 | 2005 | 2008 | 30 | [64] |
| CSE Asia | India | ADMIN2 | 574 | 2004 | 2007 | 72 | [65] |
| CSE Asia | Indonesia | ADMIN2 | 346 | 2005 | 2008 | 74 | [66] |
| CSE Asia | Iran | ADMIN2 | 283 | 2007 | 2008 | 76 | [40] |
| CSE Asia | Iraq | ADMIN2 | 11 | 2005 | 2008 | 105 | [40] |
| CSE Asia | Korea DPR | ADMIN1 | 7 | 2006 | 2008 | 169 | [67] |
| CSE Asia | Lao PDR | ADMIN2 | 139 | 2006 | 2008 | 41 | [68] |
| CSE Asia | Malaysia | ADMIN1 | 15 | 2003 | 2007 | 149 | [63] |
| CSE Asia | Myanmar | ADMIN3 | 325 | 2006 | 2008 | 45 | [67] |
| CSE Asia | Nepal | ADMIN3 | 75 | 2005 | 2008 | 44 | [69] |
| CSE Asia | Pakistan | ADMIN2 | 119 | 2005 | 2008 | 82 | [40] |
| CSE Asia | Papua New Guinea | ADMIN2 | 87 | 2005 | 2007 | 73 | [70] |
| CSE Asia | Philippines | ADMIN2 | 82 | 2004 | 2007 | 60 | [71] |
| CSE Asia | Republic of Korea | ADMIN2 | 239 | 2005 | 2008 | 20 | [72] |
| CSE Asia | Solomon Islands | ADMIN1 | 10 | 2003 | 2007 | 54 | [63] |
| CSE Asia | Sri Lanka | ADMIN2 | 25 | 2006 | 2009 | 52 | [73] |
| CSE Asia | Tajikistan | ADMIN2 | 56 | 2005 | 2008 | 50 | [74] |
| CSE Asia | Thailand | ADMIN1 | 76 | 2006 | 2008 | 82 | [75] |
| CSE Asia | Timor-Leste | ADMIN1 | 13 | 2008 | 2008 | 34 | [76] |
| CSE Asia | Turkey | ADMIN2 | 926 | 2008 | 2008 | 29 | [77] |
| CSE Asia | Vanuatu | ADMIN1 | 6 | 2003 | 2007 | 45 | [63] |
| CSE Asia | Viet Nam | ADMIN2 | 671 | 2005 | 2008 | 22 | [78] |

*Multiple requests to relevant authorities for more recent or higher spatial resolution API data were unsuccessful.

**References**
