# Supplementary material for: The International Limits and Population at Risk of Plasmodium vivax Transmission in 2009
Source: PLoS Negl Trop Dis. 2010 Aug 3;4(8):e774. doi: 10.1371/journal.pntd.0000774 (PMC2914753; doi:10.1371/journal.pntd.0000774)
Supplement: Protocol S2 — Defining the global biological limits of Plasmodium vivax transmission. Document describing more extensively two of the layers used to create the final map. (0.42 MB DOC) [file pntd.0000774.s002.doc]

**PROTOCOL S2: Defining the global biological limits of *Plasmodium vivax* transmission**

**The temperature mask**

Temperature affects many aspects of mosquito and parasite physiology [1]. One aspect, which is critical for malaria transmission, is the temperature dependence of sporogony: the time required for *Plasmodium vivax* sporozoites to develop in *Anopheles* mosquitoes. A method for estimating the duration of sporogony has been proposed, based on the number of degree-days required by the parasite to complete development [2,3], or the sum of the number of degrees in a day by which the mean temperature exceeds the minimum required for the development of sporozoites. Nikolaev [4] showed that the degree-days required for the maturation of sporozoites in an *An. maculipennis* population from Russia was 105 for *P. vivax*, 111 for *P. falciparum* and 144 for *P. malariae* and that parasite development ceased below 16 ^o^C for *P. falciparum* and *P. malariae* and below 14.5 ^o^C for *P. vivax*. The duration of sporogony (*DS*) in days can thus be calculated as:

$$DS=\frac{DD}{T-T_{th}}$$

where *DD* is the parasite species-specific number of degree-days required for sporogony, *T* is the ambient temperature and *T_th_* is the minimum temperature required for parasite development.

Figure 1 illustrates the implications of the above equation for *P. vivax*, *P. falciparum* and *P. malariae* development in *An. maculipennis* across a range of temperatures. It shows that *P. vivax* is able to develop at the lowest temperatures, followed by *P. falciparum* and *P. malariae*, thus helping explain the species-specific latitudinal and altitudinal limits of the parasites globally [5,6]. The curves never reach a true asymptote on the *y-*axis, but the duration of sporogony becomes so extended that few anophelines will survive long enough to inoculate humans and at 14.5 ^o^C *P. vivax* parasite development ceases entirely. Conversely, as temperature increases, the duration of sporogony decreases and at 30 ^o^C it can take less than ten days. At these high temperatures, the feasibility of sporogony becomes limited by parasite and vector survival, which plummet as mean temperatures rise above 32 ^o^C [7]. The duration of sporogony is dependent fundamentally on enzyme kinetics [8] and thus is widely assumed to be relatively independent of vector species. It is the interplay between the duration of sporogony and the species-specific longevity of the *Anopheles* vector that forms the basis of the temperature mask.

Synoptic mean, maximum, and minimum monthly temperature data were obtained from the WorldClim data resource in the form of 30-arcsec spatial resolution (corresponding approximately to pixels of 1 × 1 km at the equator) climate surfaces [9]. These interpolated climatologies reflect the long-term mean values for the 1950-2000 period and are available globally for each synoptic month, providing a total of 36 surfaces (mean, maximum, and minimum surfaces for each of the 12 months of the year).


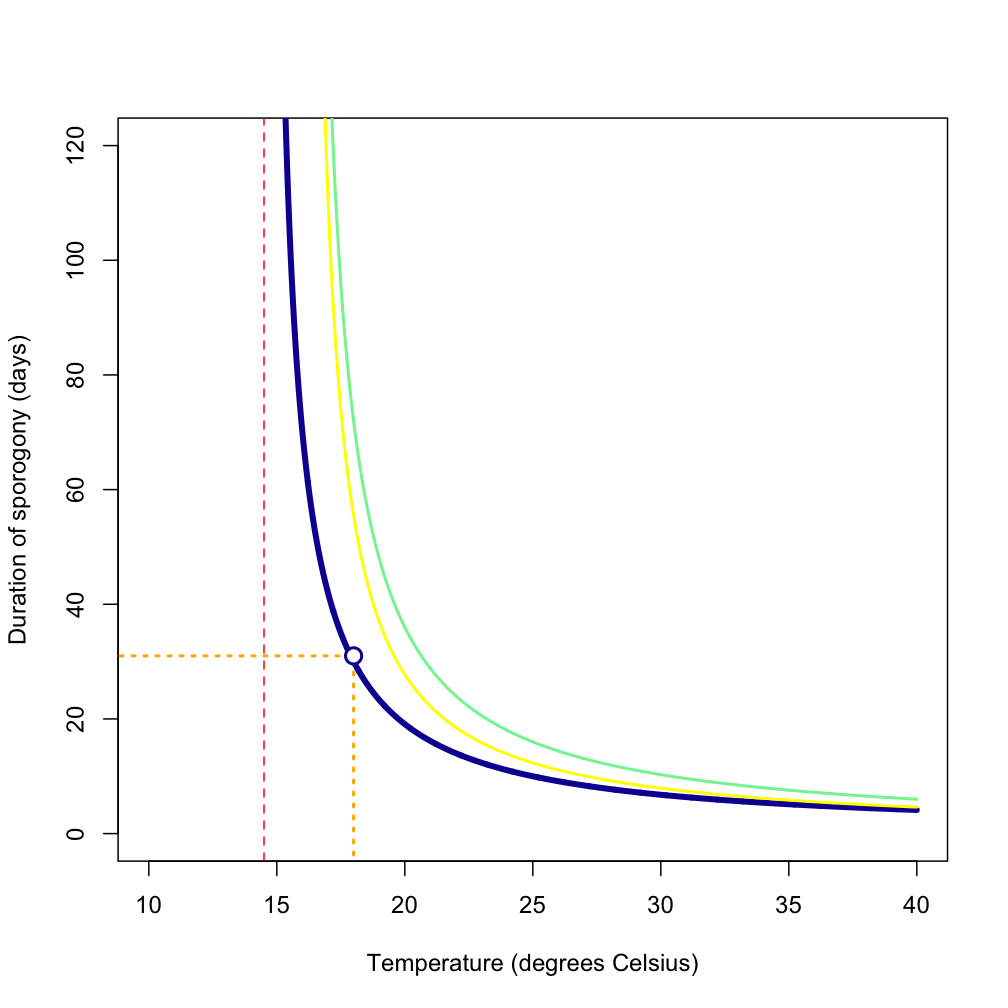


**Protocol S2, Figure 1**. The relationship between temperature and the duration of sporogony. *P. vivax* (blue), *P. falciparum* (yellow) and *P. malariae* (green). The open circle indicates the temperature below which most vectors do not outlive the length of sporogony for *P. vivax*, which corresponds to 31 days at *circa* 18 ^o^C (dotted orange lines). The dashed red line indicates the absolute temperature below which *P. vivax* development ceases (14.5 ^o^C).

The set of 12 monthly mean values available for each 1 × 1 km pixel were converted into a continuous mean temperature time-series using spline interpolation. Diurnal variation was superimposed on this time-series using a sinusoidal function with wavelength = 24 hrs. The amplitude of diurnal variation was determined as half of the difference between spline interpolated maximum and minimum temperature time series, such that the final temperature value T for a given time *t* was derived as:

$$T(t)=\frac{1}{2}(Tmx(t)-Tmn(t))\sin(\omega t+\phi)+Tmean(t)$$

Where *Tmx*, *Tmn* and *Tmean* are the values of the splined maximum, minimum and mean temperature time series, respectively; *ω* is the angular speed (π/12, for a daytime period); *t* is the time in hours; and *ϕ* is the phase, which was set to zero, thus, assuming maximum and minimum temperatures were reached at noon and midnight, respectively. For subsequent calculations, the final curve was discretised into 4,380 units, each representing a time period of two hours.

Because ambient temperatures vary constantly throughout a year, the temperature regime experienced by vectors at a given location will vary according to their emergence date. The limiting effect of temperature on sporogony was estimated at each pixel for 365 separate “cohorts” of vectors, each emerging on a different day of the year and, therefore, experiencing a unique temperature regime throughout their lifespan. The time required to reach sporogony (i.e. the accumulation of 105 degree days) was calculated for each cohort and those for which this duration exceeded the expected lifespan of the dominant vector species were considered unable to support transmission. Those pixels in which no cohorts were able to support transmission were classified as being at zero risk of transmission.

Vector lifespan was defined as 31 days since estimates of the longevity of the main dominant vectors [10] indicate that 99% of anophelines die in less than this time span. The exceptions were areas that support the longer-lived *Anopheles sergentii* and *An. superpictus*, where a lifespan of 62 days was considered more appropriate biologically [11]. Although temperature will affect other parameters of the basic reproduction rate of infection [12], including vector biting and resting habits, it seems reasonable to consider the proportion of the population surviving 31 days as the critical point of interruption of *P. vivax* transmission. To a close approximation, most vector populations would have been reduced to 1% of their original population size within 31 days. With the exception of *An. sergentii* and *An. superpictus*, it is rare for adult dominant vectors of malaria to survive longer than a month, with more than 99% of the average population dying after 31 days. The longer-lived vectors are generally those adapted to survive at higher altitudes or harsher conditions, such as *An. superpictus* and *An. sergentii*. Despite the fact that a relatively small proportion of the populations of these vectors are normally able to survive longer than one month, the number of individuals surviving might still pose a significant risk in terms of malaria transmission by being able to support parasite development at lower temperatures. After 62 days, however, most individuals of both species (>95%) would also have succumbed.

**
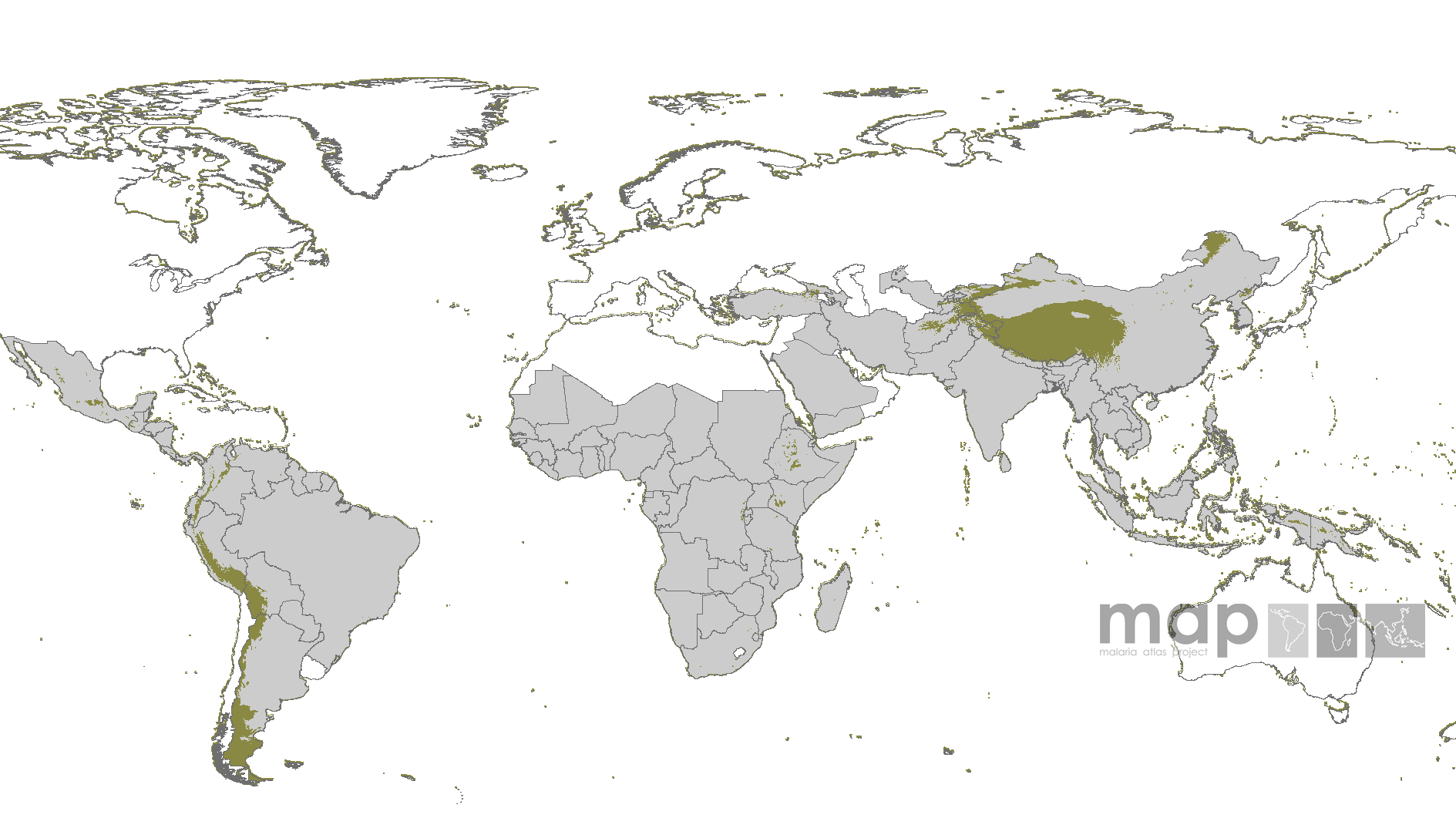
**

**Protocol S2, Figure 2**. Areas of *P. vivax* malaria endemic countries (*Pv*MECs) excluded by the temperature mask (dark green pixels). Areas climatically suitable for *P. vivax* transmission are shown in grey, and the remaining non-endemic world is shaded white.

**The aridity mask**

The ability of adult vectors to survive long enough to contribute to parasite transmission, and of their eggs and larvae to survive in sufficient numbers to sustain transmission, is dependent on the level of ambient humidity and the species-specific ability to withstand arid conditions [13-15]. These potentially limiting conditions prevail in deserts and their fringes found in malaria endemic countries. Extremely arid areas were identified using the global Land Cover product of the GlobCover project (ESA/ESA GlobCover Project, led by MEDIAS-France/POSTEL) [16]. This product is derived from data provided by the Medium Resolution Imaging Spectrometer (MERIS), on board the European Space Agency’s (ESA) ENVIronmental SATellite (ENVISAT) for the period between December 2004 and June 2006 at a spatial resolution of 300 meters. The GlobCover Land Cover product classifies the land surface into 22 classes amongst which the “bare areas” class (pixel value = 200) represents desert biomes. The land cover layer was resampled to 1 × 1 km spatial resolution using a majority filter to match the other layers and was overlaid on top of the temperature mask. The aridity mask worked in a step-wise fashion by which risk was down-regulated one class from its initial value (i.e. stable to unstable and unstable to no risk). Therefore, the only areas where risk was excluded were those where the *Pv*API layer had already defined unstable risk of malaria.


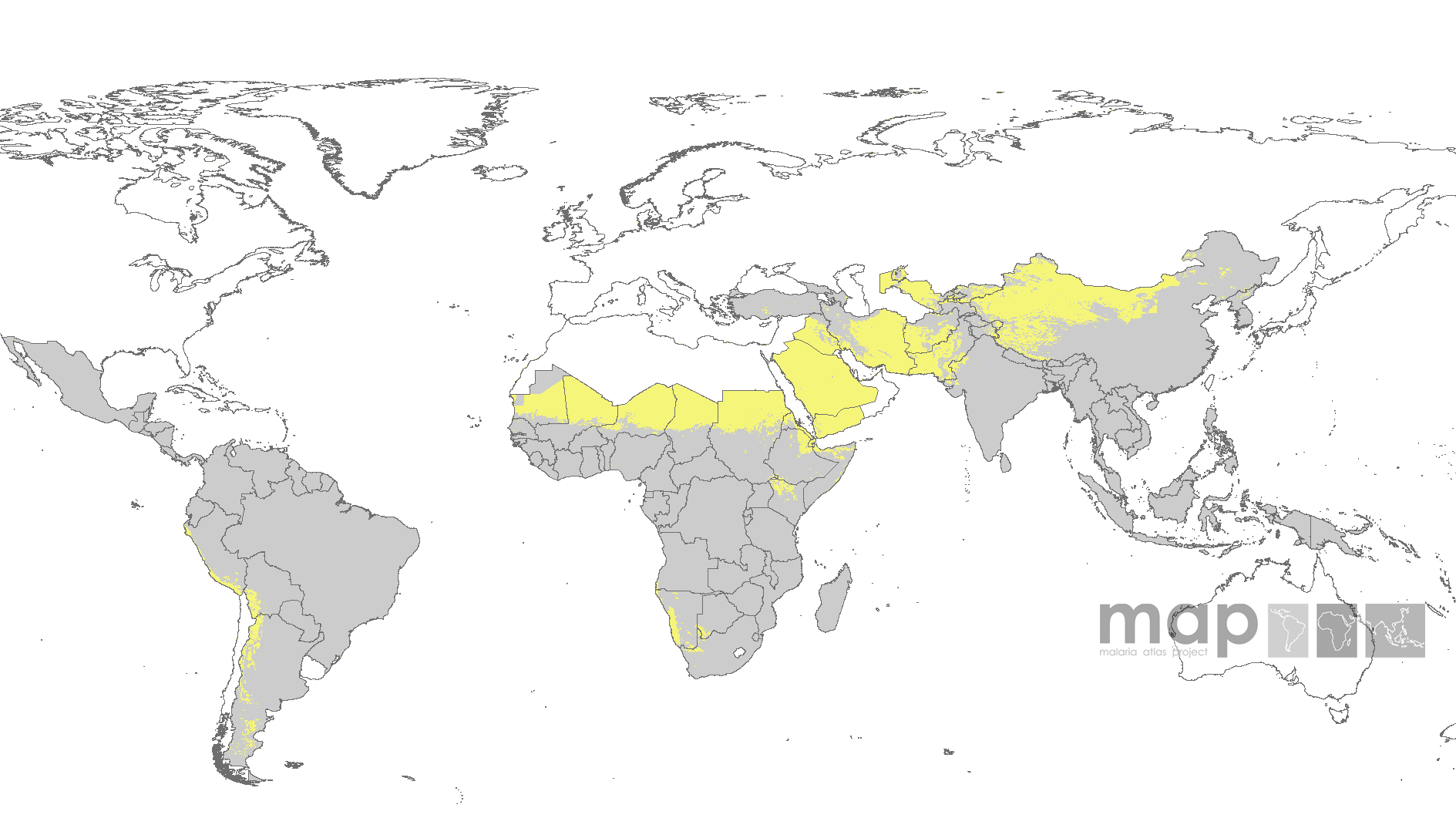


**Protocol S2, Figure 3**. The “bare areas” class of the GlobCover Land Cover product (yellow pixels). The mask is depicted within the territories of *Pv*MECs.

**References**

1. Clements AN (1999) The Biology of Mosquitoes. Wallingford, UK: CABI Publishing. 740 p.

2. Detinova TS (1962) Age grouping methods in *Diptera* of medical importance with special reference to some vectors of malaria. Geneva: World Health Organization.

3. Moshkovsky SD (1946) The dependence upon temperature of the speed of development of malaria *plasmodia* in the mosquito [in Russian]. Med Parazitol (Mosk) 15: 19.

4. Nikolaev BP (1935) On the influence of temperature on the development of malaria *plasmodia* inside the mosquito. Leningrad Pasteur Institute of Epidemiology and Bacteriology.

5. Lysenko AJ, Beljaev AE (1969) An analysis of the geographical distribution of *Plasmodium ovale*. Bull World Health Organ 40: 383-394.

6. Lysenko AY, Semashko IN (1968) Geography of Malaria: a medico-geographic profile of an ancient disease [in Russian]. In: Lebedew AW, editor. Medicinskaja Geografija. Moscow: Academy of Sciences. pp. 25-146.

7. Muirhead-Thompson RC (1951) Mosquito behaviour in relation to malaria transmission and control in the tropics. London: Edward Arnold & Co.

8. Sharpe PJ, DeMichele DW (1977) Reaction kinetics of poikilotherm development. J Theor Biol 64: 649-670.

9. Hijmans RJ, Cameron SE, Parra JL, Jones PG, Jarvis A (2005) Very high resolution interpolated climate surfaces for global land areas. Int J Climatol 25: 1965-1978.

10. Kiszewski A, Mellinger A, Spielman A, Malaney P, Sachs SE, et al. (2004) A global index representing the stability of malaria transmission. Am J Trop Med Hyg 70: 486-498.

11. Guerra CA, Gikandi PW, Tatem AJ, Noor AM, Smith DL, et al. (2008) The limits and intensity of *Plasmodium falciparum* transmission: implications for malaria control and elimination worldwide. PLoS Med 5: e38.

12. Rogers DJ, Randolph SE (2006) Climate change and vector-borne diseases. Adv Parasitol 62: 345-381.

13. Shililu JI, Grueber WB, Mbogo CM, Githure JI, Riddiford LM, et al. (2004) Development and survival of *Anopheles gambiae* eggs in drying soil: influence of the rate of drying, egg age, and soil type. J Am Mosq Control Assoc 20: 243-247.

14. Omer SM, Cloudsley-Thompson JL (1970) Survival of female *Anopheles gambiae* Giles through a 9-month dry season in Sudan. Bull World Health Organ 42: 319-330.

15. Omer SM, Cloudsley-Thomson JL (1968) Dry season biology of *Anopheles gambiae* Giles in the Sudan. Nature 217: 879-880.

16. Bicheron P, Defourny P, Brockmann C, Schouten L, Vancutsem C, et al. (2008) GLOBCOVER: Products Description and Validation Report. Tolouse: MEDIAS-France.
