# Supplementary material for: The International Limits and Population at Risk of Plasmodium vivax Transmission in 2009
Source: PLoS Negl Trop Dis. 2010 Aug 3;4(8):e774. doi: 10.1371/journal.pntd.0000774 (PMC2914753; doi:10.1371/journal.pntd.0000774)
Supplement: Protocol S3 — Risk modulation based upon medical intelligence. Document describing more extensively one of the layers used to create the final map. (0.36 MB DOC) [file pntd.0000774.s003.doc]

**PROTOCOL S3: Risk modulation based upon medical intelligence**

**Urban transmission**

Urban areas are less malarious than the surrounding rural environments due to the distinct ecological conditions presented by man-made environments [1,2]. The extent to which transmission is reduced will vary according to the local *Anopheles* species. Urbanization has been shown to reduce malaria transmission, measured by the entomological inoculation rate, by an order of magnitude across Africa, due to reduced vector diversity and density, as well as lower anopheline survival, biting and sporozoite rates in urban versus rural areas [1]. *Anopheles darlingi*, the main malaria vector in America, has shown itself to be similarly unsuited to urban environments [3].

Urban malaria transmission is more entrenched in the Indian subcontinent because of the presence of *An. stephensi* and, to a lesser extent, *An. culicifacies*, both recognised urban malaria vectors [4]. No malaria vector is better adapted to urban environments than *An. stephensi*, and this is due to its ability to breed in all types of artificial collections of water, such as wells, pits, tanks and drains [5]. *Anopheles culicifacies* is less resilient to man-made environments and is particularly affected by pollution of water sources [5,6]. Importantly, the vector densities and sporozoite rates of both these species have been shown to decrease from peri-urban to urban areas [5,7,8]. Despite this decrease, it is estimated that approximately 8% of reported malaria cases in India come from urban areas [9], with incidence often surpassing the stable risk threshold. Reported annual parasite incidence (API) estimates amongst 86 cities across India in 1993 ranged from 0 to 51.85 cases per 1,000 people per annum (p.a.), with a median of 0.97 [10]. Seventy of these cities would have been classified as supporting stable transmission according to the API threshold used in this paper (i.e. API ≥0.1 case per 1,000 people p.a.). In 2004, the API in an impoverished area located in the outskirts of Kolkata was measured at 1.5 cases per 1,000 residents p.a., with the majority (97%) due to *P. vivax* [11]. Since *An. culicifacies* seems to be more affected by the process of urbanisation, it was assumed that urban malaria transmission is maintained mainly by *An. stephensi* (Figure) as defined by the rules of risk modulation described below.


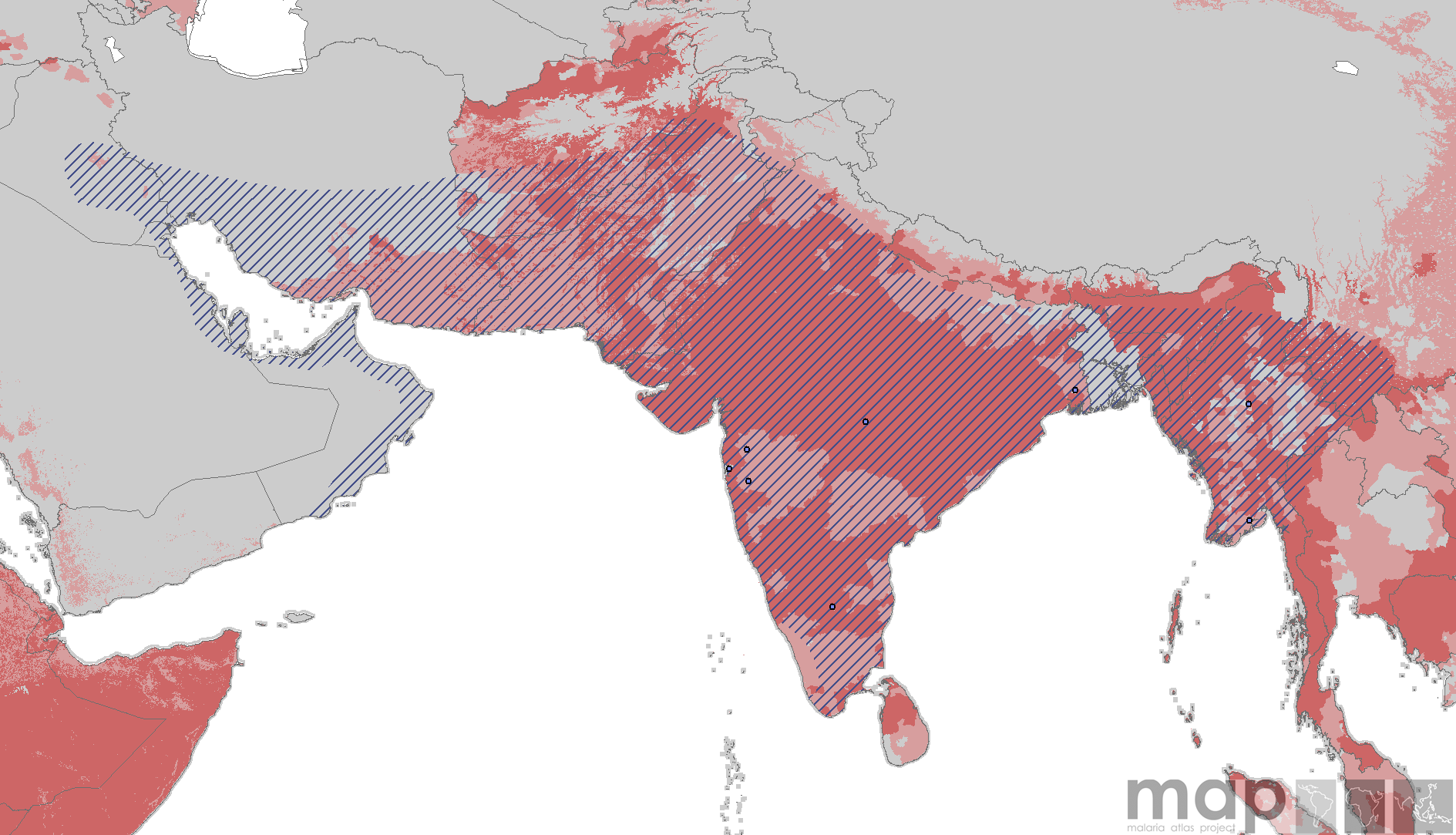


**Protocol S3, Figure.** Distribution of *An. stephensi* [12] (hatched area) and location of the eight cities falling within this range (light blue circles) overlaid on the *Pv*API/biological masks-defined *P. vivax* limits of transmission.

**Risk modulation in specified urban areas**

There are 59 cities cited as being malaria free in the two sets of international travel and health guidelines consulted [13,14] (Table 1). In addition, urban areas in China, the Philippines and Indonesia (specifically those located in Sumatra, Kalimantan, Nusa Tenggara Barat and Sulawesi) are said to be malaria free. This is obviously not a comprehensive list of malaria free cities but rather one restricted to main destinations of interest to travellers. Specific cities were geo-positioned and their urban extents were identified using the Global Rural Urban Mapping Project (GRUMP) urban extents layer [15]. In China, the Philippines and specified areas of Indonesia all urban extents were identified and mapped. The resulting layer was overlaid on the *Pv*API layer and biological masks to identify the underlying risk of malaria. Those cities falling within the range of *An. stephensi* [12] were also identified.

Of the 59 specified cities, 17 are in areas where malaria transmission is absent as defined by the *Pv*API layer and the biological masks (e.g. highland areas). The urban extents of the remaining 42 cities cover areas defined as unstable or stable transmission or both (Table 1). Only eight of these cities fall within the range of *An. stephensi*: six in India (Bangalore, Kolkata, Mumbai, Nagpur, Nashik and Pune) and two in Myanmar (Mandalay and Yangon; Figure). In addition, cities in south-western Yunnan, China, also fall in areas inhabited by this vector.

For all cities falling outside the range of *An. stephensi*, risk was classified as absent throughout their urban extents. For those cities falling within the range of *An. stephensi*, risk was assumed to be one level lower than the surrounding risk defined by *Pv*API data and the biological masks. This is to allow for the potential transmission of malaria by *An. stephensi* combined with the transmission reducing effects of urban areas [5,7,8].

**Protocol S3, Table 1.** Cities cited as being malaria free by the sources consulted [13,14]. Defined risk refers to the malaria risk categories defined by the *Pv*API layer and biological masks; note that urban extents often cover more than one category. Modified risk refers to the new malaria risk categories assigned according to the rules described in the text. Cities where the defined risk was “free” were not affected by these rules.

| **Country** | **City** | **Defined risk** | **Modified risk** |
| --- | --- | --- | --- |
| Azerbaijan | Baku | Free, unstable | Free |
| Bangladesh | Dhaka | Free | - |
| Belize | Belize | Unstable | Free |
| Bolivia | La Paz | Free | - |
| Botswana | Gaborone | Free | - |
| Cambodia | Phnom Penh | Free, unstable | Free |
| Colombia | Bogota | Free, unstable | Free |
| Colombia | Cartagena | Free, unstable | Free |
| Costa Rica | Puerto Limon | Unstable | Free |
| Ecuador | Guayaquil | Unstable, stable | Free |
| Ecuador | Quito | Free | - |
| Eritrea | Asmara | Stable | Free |
| Ethiopia | Addis Ababa | Stable, free | Free |
| French Guiana | Cayenne | Free | - |
| Georgia | Tblisi | Unstable, free | Free |
| Guatemala | Antigua | Free | - |
| Guatemala | Guatemala | Free | - |
| Honduras | San Pedro Sula | Unstable | Free |
| Honduras | Tegucigalpa | Unstable, free | Free |
| India | Bangalore | Stable | Unstable |
| India | Kolkata | Unstable, stable | Free, unstable |
| India | Mumbai | Stable, unstable | Unstable, free |
| India | Nagpur | Stable | Unstable |
| India | Nasik | Unstable | Free |
| India | Pune | Unstable | Free |
| Indonesia | Jakarta | Free | - |
| Iraq | Baghdad | Free | - |
| Iraq | Ramadi | Free | - |
| Iraq | Tikrit | Free | - |
| Kenya | Nairobi | Stable | Free |
| Kyrgyzstan | Bishkek | Free | - |
| Laos | Vientiane | Free, unstable | Free |
| Myanmar | Mandalay | Free, stable, unstable | Free, unstable |
| Myanmar | Yangon | Unstable | Free |
| Nepal | Kathmandu | Free | - |
| Nicaragua | Managua | Unstable | Free |
| Panama | Panama | Unstable | Free |
| Peru | Cuzco | Free | - |
| Saudi Arabia | Jeddah | Unstable | Free |
| Saudi Arabia | Mecca | Unstable | Free |
| Saudi Arabia | Medina | Unstable | Free |
| Saudi Arabia | Riyadh | Free | - |
| Saudi Arabia | Ta'if | Unstable | Free |
| Suriname | Paramaribo | Free | - |
| Thailand | Bangkok | Free, unstable | Free |
| Thailand | Chiang Mai | Stable | Free |
| Thailand | Chiang Rai | Unstable | Free |
| Thailand | Koh Phangan | Stable | Free |
| Thailand | Koh Samui | Stable | Free |
| Thailand | Pattaya | Unstable | Free |
| Viet Nam | Can Tho | Free, unstable | Free |
| Viet Nam | Da Nang | Unstable | Free |
| Viet Nam | Haiphong | Free | - |
| Viet Nam | Hanoi | Free, unstable | Free |
| Viet Nam | Ho Chi Minh City | Unstable | Free |
| Viet Nam | Hue | Free, unstable | Free |
| Viet Nam | Nha Trang | Free, unstable | Free |
| Viet Nam | Qui Nhon | Unstable | Free |
| Yemen | Sana’a | Unstable | Free |

**Risk exclusion in administrative areas**

Some sub-national administrative areas and territories are listed as being malaria free by the international travel and health guidelines consulted [13,14]. These are shown in Table 2. Such territories were mapped using the GAUL data set [16] and risk within them was assigned a malaria free category, if not already classified as such by the *Pv*API layer and the biological masks. In addition to the territories listed in Table 2, the island of Socotra, in Yemen, has not reported cases since 2005 after malaria elimination activities were initiated in 2000 [17]; this island was assumed to be malaria free. Two further exclusions were those of the island of Aneityum, in Vanuatu [18], and the Angkor Watt area, in Cambodia (corresponding to two districts in Siem Reap province), which were classified as malaria free following personal communication with malaria experts in these countries.

**Protocol S3, Table 2.** Administrative areas defined as being malaria free by international travel and health guidelines.

| **Country** | **Administrative Units** |
| --- | --- |
| Ecuador | Galapagos |
| French Guiana | Devil's Island |
| Mauritania | Adrar, Dakhlet-Nouadhibou, Inchiri and Tiris-Zemmour regions |
| Philippines | Aklan, Albay, Benguet, Bilaran, Bohol, Camiguin, Capiz, Catanduanes, Cavite, Cebu, Guimaras, Iloilo, Northern Leyte, Southern Leyte, Marinduque, Masbate, Eastern Samar, Northern Samar, Western Samar, Sequijor, Sorsogon, Surigao Del Norte and metropolitan Manila |
| Sri Lanka | Colombo, Galle, Gampaha, Kalutara, Matara, and Nuwara Eliya |
| Venezuela | Margarita Island |

**References**
