# Supplementary material for: The International Limits and Population at Risk of Plasmodium vivax Transmission in 2009
Source: PLoS Negl Trop Dis. 2010 Aug 3;4(8):e774. doi: 10.1371/journal.pntd.0000774 (PMC2914753; doi:10.1371/journal.pntd.0000774)
Supplement: Protocol S4 — Country level area and population at risk of Plasmodium vivax malaria in 2009. Country-level table of the estimated area and populations at risk of P. vivax malaria in 2009 (0.16 MB DOC) [file pntd.0000774.s004.doc]

**PROTOCOL S4: Country level area and population at risk of *Plasmodium vivax* malaria in 2009.**

**Protocol S4, Table.** Area and population at risk (PAR) of *Plasmodium vivax* malaria in 2009. Unstable and stable risk correspond to *Pv*API <0.1 case per 1,000 people per annum and *Pv*API ≥0.1 case per 1,000 per annum, respectively. Total estimated country areas and populations are also listed.

| **Region/Country** | **Risk area (km^2^)** | | **Country area (km^2^)** | **PAR** | | **Country population*** |
| --- | --- | --- | --- | --- | --- | --- |
|  | **Unstable** | **Stable** |  | **Unstable** | **Stable** |  |
| ***Africa*+** |  |  |  |  |  |  |
| Angola | 9,881 | 1,245,119 | 1,255,019 | 10,709 | 468,918 | 20,251,918 |
| Benin | 16 | 116,287 | 116,303 | 12 | 49,499 | 10,296,822 |
| Botswana | 1,019 | 399,723 | 580,821 | 401 | 144,230 | 1,955,923 |
| Burkina Faso | 2,520 | 272,542 | 275,062 | 83 | 184 | 17,699,813 |
| Burundi | 0 | 25,569 | 27,127 | 0 | 110,160 | 11,522,427 |
| Cameroon | 984 | 467,708 | 469,305 | 415 | 201,403 | 20,554,675 |
| Central African Republic | 70 | 624,173 | 624,244 | 342 | 223,540 | 4,169,888 |
| Chad | 692,215 | 585,050 | 1,277,265 | 158,102 | 699,964 | 12,242,955 |
| Comoros | 0 | 1,620 | 1,688 | 0 | 62,784 | 687,650 |
| Congo | 0 | 343,876 | 343,876 | 0 | 41,396 | 4,280,934 |
| Côte d'Ivoire | 29 | 323,945 | 323,974 | 0 | 0 | 22,108,971 |
| Democratic Republic of the Congo | 53 | 2,330,763 | 2,343,648 | 43 | 1,743,141 | 75,067,116 |
| Djibouti | 14,691 | 7,132 | 21,823 | 396,705 | 250,369 | 874,932 |
| Equatorial Guinea | 0 | 26,886 | 27,132 | 0 | 30 | 549,018 |
| Eritrea | 48,635 | 72,339 | 121,107 | 717,189 | 1,785,801 | 5,826,613 |
| Ethiopia | 75,318 | 1,003,891 | 1,136,443 | 2,019,121 | 39,196,948 | 99,105,393 |
| Gabon | 0 | 266,519 | 266,519 | 0 | 142 | 1,385,000 |
| Ghana | 441 | 239,865 | 240,306 | 0 | 0 | 27,621,035 |
| Guinea | 194 | 246,211 | 246,404 | 0 | 0 | 11,173,309 |
| Guinea-Bissau | 42 | 34,105 | 34,147 | 0 | 0 | 1,786,467 |
| Kenya | 63,820 | 507,261 | 588,770 | 19,728 | 493,366 | 42,692,919 |
| Liberia | 1 | 96,484 | 96,485 | 0 | 0 | 4,557,120 |
| Madagascar | 0 | 592,050 | 594,641 | 0 | 5,786,508 | 22,444,977 |
| Malawi | 0 | 118,196 | 118,779 | 0 | 5,168 | 18,297,086 |
| Mali | 728,852 | 529,651 | 1,258,503 | 11,904 | 8,825 | 17,284,856 |
| Mauritania | 311,053 | 187,083 | 1,044,247 | 25,458 | 4,322 | 3,264,333 |
| Mozambique | 50 | 791,402 | 791,668 | 56 | 631,892 | 26,347,358 |
| Namibia | 344,002 | 380,889 | 828,351 | 442,115 | 259,280 | 2,094,155 |
| Niger | 848,520 | 341,751 | 1,190,272 | 86,413 | 307,755 | 16,104,855 |
| Nigeria | 2,093 | 913,117 | 915,209 | 7,920 | 2,503,416 | 167,795,904 |
| Rwanda | 0 | 21,213 | 25,369 | 0 | 113,328 | 13,642,123 |
| São Tomé and Príncipe | 0 | 991 | 1,015 | 0 | 0 | 185,888 |
| Saudi Arabia | 13,799 | 0 | 1,934,670 | 168,216 | 0 | 26,670,007 |
| Senegal | 1,368 | 196,554 | 197,922 | 0 | 0 | 13,506,729 |
| Sierra Leone | 4 | 72,821 | 72,825 | 0 | 0 | 6,434,393 |
| Somalia | 59,564 | 577,663 | 637,382 | 370,774 | 1,753,062 | 9,844,542 |
| South Africa | 111,659 | 134,272 | 1,224,335 | 2,995,850 | 648,523 | 52,349,878 |
| Sudan | 1,063,504 | 1,453,243 | 2,516,856 | 3,746,386 | 12,204,942 | 47,369,593 |
| Swaziland | 3,176 | 3,520 | 17,354 | 15,555 | 21,246 | 1,184,815 |
| Tanzania (United Republic of) | 943 | 937,790 | 947,189 | 6 | 126,722 | 50,174,891 |
| The Gambia | 33 | 10,838 | 10,870 | 0 | 0 | 1,973,490 |
| Togo | 26 | 57,383 | 57,409 | 0 | 20 | 8,008,571 |
| Uganda | 1,142 | 238,095 | 242,847 | 904 | 883,170 | 35,071,856 |
| Yemen | 412,901 | 38,706 | 456,112 | 8,891,688 | 6,238,753 | 27,655,798 |
| Zambia | 0 | 755,369 | 755,369 | 0 | 293,050 | 12,562,258 |
| Zimbabwe | 0 | 391,045 | 392,731 | 0 | 606,447 | 14,896,994 |
| ***America*** |  |  |  |  |  |  |
| Argentina | 24,833 | 30,319 | 2,786,654 | 853,692 | 365,876 | 40,005,789 |
| Belize | 4 | 22,250 | 22,377 | 28 | 254,514 | 316,782 |
| Bolivia | 105,376 | 486,272 | 1,090,422 | 880,144 | 2,822,094 | 9,782,927 |
| Brazil | 502,618 | 4,398,750 | 8,535,550 | 35,369,880 | 15,020,891 | 203,072,862 |
| Colombia | 100,204 | 860,662 | 1,145,439 | 16,470,110 | 10,600,006 | 48,914,664 |
| Costa Rica | 21,613 | 3,140 | 51,549 | 943,816 | 107,822 | 4,937,871 |
| Ecuador | 33,745 | 137,246 | 257,911 | 2,280,715 | 3,841,676 | 14,822,144 |
| El Salvador | 15,537 | 0 | 20,943 | 4,229,158 | 0 | 7,444,007 |
| French Guiana | 0 | 81,456 | 83,927 | 0 | 143,874 | 206,174 |
| Guatemala | 1,579 | 80,321 | 109,704 | 151,260 | 5,866,131 | 15,059,418 |
| Guyana | 35,885 | 175,833 | 211,745 | 118,213 | 544,408 | 662,621 |
| Honduras | 17,206 | 77,296 | 113,449 | 1,610,163 | 3,152,128 | 8,278,068 |
| Mexico | 183,888 | 103,830 | 1,959,530 | 9,728,301 | 2,675,986 | 111,991,697 |
| Nicaragua | 14,625 | 113,978 | 129,797 | 1,742,011 | 3,024,581 | 5,752,310 |
| Panama | 10,858 | 43,251 | 75,517 | 927,280 | 740,580 | 3,836,817 |
| Paraguay | 5,928 | 17,905 | 401,592 | 164,949 | 993,585 | 6,954,457 |
| Peru | 73,909 | 719,696 | 1,294,642 | 4,496,082 | 3,956,235 | 28,505,600 |
| Suriname | 0 | 121,244 | 146,970 | 0 | 5,289 | 445,294 |
| Venezuela | 220,570 | 613,887 | 918,069 | 19,030,961 | 4,701,225 | 29,388,111 |
| ***Central Asia*** |  |  |  |  |  |  |
| Cambodia | 12,998 | 166,038 | 182,555 | 4,651,810 | 12,913,783 | 19,396,849 |
| China | 2,518,353 | 298,769 | 9,433,834 | 582,648,764 | 43,418,786 | 1,649,808,114 |
| Korea, Democratic People's Republic of | 5,178 | 94,487 | 122,645 | 921,299 | 20,969,013 | 23,742,896 |
| Korea, Republic of | 4,831 | 5,838 | 99,025 | 1,051,732 | 2,054,494 | 49,715,677 |
| Lao People's Democratic Republic | 56,680 | 21,695 | 231,195 | 2,349,546 | 482,180 | 9,145,551 |
| Myanmar | 177,188 | 393,799 | 671,205 | 22,932,011 | 25,169,538 | 60,813,797 |
| Thailand | 212,629 | 206,152 | 517,052 | 25,756,838 | 18,333,302 | 70,404,317 |
| Viet Nam | 168,718 | 90,443 | 330,652 | 53,959,109 | 5,832,705 | 103,624,061 |
| ***East Asia*** |  |  |  |  |  |  |
| Indonesia | 459,108 | 1,248,420 | 1,902,836 | 110,683,213 | 64,684,480 | 291,296,656 |
| Malaysia | 96,323 | 235,053 | 331,961 | 23,030,870 | 7,841,060 | 30,934,920 |
| Papua New Guinea | 52,909 | 361,413 | 466,397 | 385,415 | 4,318,809 | 5,817,971 |
| Philippines | 76,718 | 152,442 | 297,556 | 26,671,130 | 30,965,311 | 101,239,624 |
| Solomon Islands | 0 | 25,512 | 25,532 | 0 | 583,757 | 584,056 |
| Timor-Leste | 0 | 14,813 | 14,984 | 0 | 1,203,758 | 1,224,513 |
| Vanuatu | 61 | 12,072 | 12,295 | 1,177 | 264,013 | 269,934 |
| ***West Asia*** |  |  |  |  |  |  |
| Afghanistan | 215,358 | 232,905 | 643,052 | 12,303,262 | 19,347,189 | 37,929,163 |
| Azerbaijan | 49,929 | 1,399 | 86,489 | 4,033,912 | 45,693 | 8,679,818 |
| Bangladesh | 0 | 42,783 | 139,479 | 0 | 38,363,259 | 176,487,357 |
| Bhutan | 2,571 | 13,850 | 37,817 | 206,440 | 1,875,557 | 3,189,189 |
| Georgia | 4,593 | 3,099 | 69,832 | 297,759 | 189,784 | 4,302,474 |
| India | 787,541 | 2,183,691 | 3,164,715 | 473,730,503 | 720,599,622 | 1,254,065,755 |
| Iran | 233,161 | 21,285 | 1,625,947 | 3,124,312 | 473,303 | 77,831,473 |
| Iraq | 18,181 | 0 | 437,266 | 2,323,368 | 0 | 29,276,722 |
| Kyrgyzstan | 33,234 | 0 | 199,351 | 1,934,151 | 0 | 5,502,695 |
| Nepal | 52,356 | 33,196 | 147,868 | 18,885,986 | 12,742,832 | 38,632,646 |
| Pakistan | 532,551 | 220,765 | 879,064 | 124,302,142 | 45,938,682 | 189,933,821 |
| Sri Lanka | 35,653 | 22,478 | 66,607 | 8,125,459 | 3,443,213 | 19,855,760 |
| Tajikistan | 22,553 | 21,419 | 142,298 | 3,093,964 | 1,936,941 | 6,843,874 |
| Turkey | 15,246 | 3,740 | 782,223 | 1,268,928 | 284,329 | 81,454,747 |
| Uzbekistan | 4,319 | 0 | 449,909 | 307,172 | 0 | 27,427,706 |
|  |  |  |  |  |  |  |
| ***World*** | **12,029,937** | **32,195,600** | **69,516,818** | **1,628,063,132** | **1,220,960,598** | **5,911,385,997** |

*The Global Rural Urban Mapping Project gridded population database *beta* version population estimates for 2000 are derived through adjustment to United Nations national population total estimates made in the 2004 edition of the World Populations Prospects report. Through the application of separate 2000-2005 and 2005-2010 urban and rural growth rates estimated by the most recent edition available at the time of writing of the United Nations World Urbanization Prospects (2007 edition) to obtain a 2009 population surface, the estimated population totals show some deviations from national population totals estimated by the most recent edition of the UN World Population Prospects report (2008), due to the differences in estimates and methods used between the different reports.
